# Evaluation of microneedles-assisted in situ depot forming poloxamer gels for sustained transdermal drug delivery

**DOI:** 10.1007/s13346-019-00617-2

**Published:** 2019-01-23

**Authors:** Samiullah Khan, Muhammad Usman Minhas, Ismaiel A. Tekko, Ryan F. Donnelly, Raghu Raj Singh Thakur

**Affiliations:** 1 Faculty of Pharmacy and Alternative Medicine, The Islamia University of Bahawalpur, Bahawalpur, Punjab 63100 Pakistan; 2 School of Pharmacy, Medical Biology Centre, Queen’s University Belfast, 97 Lisburn Road, Belfast, BT9 7BL UK

**Keywords:** Microneedles, Poloxamers, Microporation, In situ depots, Transdermal, Confocal microscopy

## Abstract

In this study, for the first time, we have reported a sustained transdermal drug delivery from thermoresponsive poloxamer depots formed within the skin micropores following microneedle (MN) application. Firstly, we have investigated the sol–gel phase transition characteristics of poloxamers (PF®127, P108, and P87) at physiological conditions. Rheological measurements were evaluated to confirm the critical gelation temperature (CGT) of the poloxamer formulations with or without fluorescein sodium (FS), as a model drug, at various concentrations. Optimized poloxamer formulations were subjected to in vitro release studies using a vial method. Secondly, polymeric MNs were fabricated using laser-engineered silicone micromolds from various biocompatible polymeric blends of Gantrez S-97, PEG 10000, PEG200, PVP K32, and PVP K90. The MN arrays were characterized for mechanical strength, insertion force determination, in situ dissolution kinetics, moisture content, and penetration depth. The optimized MN arrays with good mechanical strength and non-soluble nature were used to create micropores in the neonatal porcine skin. Microporation in neonatal porcine skin was confirmed by dye-binding study, skin integrity assessment, and histology study. Finally, the in vitro delivery of FS from optimized poloxamer formulations was conducted across non-porated vs microporated skin samples using vertical Franz diffusion cells. Results concluded that permeation of FS was sustained for 96 h across the MN-treated skin samples containing in situ forming depot poloxamer formulations compared to non-microporated skin which sustained the FS delivery for 72 h. Confocal microscopic images confirmed the distribution of higher florescence intensity of FS in skin tissues after permeation study in case of MN-treated skin samples vs intact skin samples.

**Electronic supplementary material:**

The online version of this article (10.1007/s13346-019-00617-2) contains supplementary material, which is available to authorized users.

## Introduction

Skin being a large and accessible organ is considered an attractive route for the administration of a wide variety of drug molecules, proteins, and vaccines. It also plays an important role as a barrier against many unwanted and foreign substances [1, 2]. Conventional transdermal delivery through skin provides a promising alternative to parenteral and oral delivery and provides many advantages such as painless delivery, bypassing first-pass metabolism, patient accessibility, and compliance [3, 4]. Despite the multiple advantages of this route, it also encountered some problems of poor permeability and low bioavailability; therefore, to date, approximately 20 active pharmaceutical agents were delivered through this route mostly in the form of transdermal patches that are commercially available. Due to the structural components of the skin, transdermal drug delivery remained a challenging task [5]. The *stratum corneum* (SC) layer of the skin, the outermost lipophilic skin layer (10–15 μm) composed of dead keratinocytes held together, has been proved to be the main physical barrier for drug flux across the skin [6, 7].

A number of strategies have been adopted by the researchers to bypass the SC barrier to enhance and sustain drug delivery via this route [8]. However, in the case of parenteral sustained release formulations, some issues remain unaddressed such as avoidance of burst release, toxicity issues, and variance in implant microstructure and shape thus limiting their practical use [9, 10]. Moreover, enhancement techniques that has also been widely explored to improve permeation and/or overcome the physical barrier to drug delivery such as iontophoresis (electrical energy), microneedles (MNs), laser ablation, sonophoresis (ultrasound), radiofrequency ablation, microdermabrasion, and thermal ablation have their own limitations [11–13].

MNs (15–2000 μm in height) are attractive micron-sized minimally invasive third-generation devices which have been developed and utilized over a decade ago. MNs have been extensively investigated for a large range of molecules to enhance their transdermal/intradermal delivery [14–16]. MNs bypass the SC layer to penetrate into the epidermis layer leading to creation of hydrophilic, interstitial fluid–filled conduits (microchannels/micropores) of micron-sized in the skin that goes deep enough to overcome the physical skin barrier but avoid contact with the nerve endings in the dermis layer [1]. These microchannels have the ability enough to allow the passage of loaded host molecules of variable size, e.g., cosmeceuticals [15], vaccines [16], monoclonal antibody [17], proteins and peptides [17, 18], and small molecules enough to evade skin irritation, damage, and infection [19, 20].

MNs can be fabricated by using different types of materials (e.g., stainless steel, silicon, glass, maltose, titanium, and polymers) and in a variety of designs (coated, hollow, solid, dissolvable), geometries to overcome SC and deliver drug to the target site in the skin or eye [4, 21–28]. Coated MNs have been investigated to allow the delivery of various drugs, and micro- and nanoparticles via transdermal route [23–26]. However, coated MNs are ideal for highly potent drugs as they provide a tiny surface area for coating [23]. On the other hand, hollow MNs made up of typically glass, metals, ceramics, and silicon deliver the drugs either by diffusion process or by bore opening through pressure [29]. However, clogging and loss of volume can occur during hollow MN application [30–32].

Dissolving polymeric MNs have shown promising strategy for transdermal drug and gene delivery [23, 33]. Dissolving MNs have been fabricated from polymers and biopolymers maltose, trehalose, polyvinylpyrrolidone (PVP), sucrose, polyglycolic acid (PGA), poly (vinylpyrrolidone-co-methacrylic acid) (PVPMAA) polylactic-co-glycolic acid (PLGA), sodium hyaluronate, poly (methyl vinyl ether-maleic anhydride), and chondroitin sulfate [34–36]. However, these types of MNs must have enough mechanical strength to create the microchannels in the skin.

Thermoresponsive polymers provide in situ forming gels upon injection to provide sustained delivery without undergoing invasive surgical procedure [37]. Among these poloxamers or pluronics, a PEO–PPO–PEO tri-block copolymers represent a series of central chain of polyoxypropylene (PPO) and two identical lateral hydrophilic chains of polyoxyethylene (PEO) [38, 39]. They have been widely used as in situ forming carrier due to their unique sol–gel transition behavior in aqueous solution in response to temperature change at skin (32 °C) and body temperature (37 °C) [1]. At room temperature (< 30 °C), the aqueous solutions remain in sol state; however, at certain temperature (> LCST, 32 °C) and concentration, it converts into gel form forming a depot system [40]. Pluronics are FDA-approved biocompatible polymer for oral, injectable, topical, inhalation, and ophthalmic preparation [3, 41]. Recently, Sivaraman et al. [3] reported the formation of in situ forming hydrogel MNs using Pluronic PF127 loaded with a model drug for sustained delivery. However, to date, no MN-assisted in situ forming depots from thermoresponsive poloxamer materials have been reported.

**Fig. 1:**
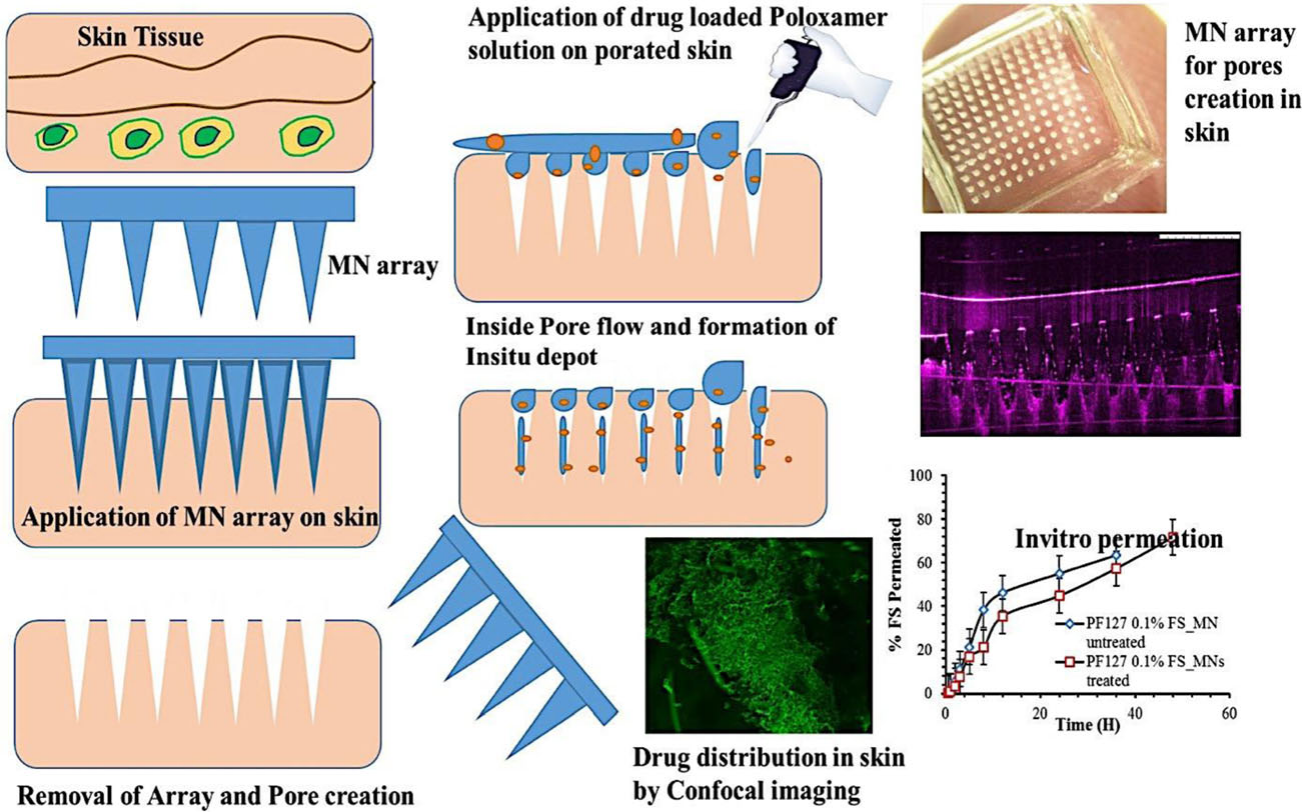
Schematic illustration of formation and drug delivery fromin situ depots of poloxomer gels formed in themicropores created by MN application. Transition loaded into MN pores and formation of in situ gels at skin temperature (32 °C)

For the first time, in this study, we reported the formation of depots of thermoresponsive poloxamers within the micropores of the skin tissues using MNs to sustain transdermal drug delivery. Briefly, MNs were fabricated from different polymers with varying heights and characterized for its mechanical strength, moisture content, and in situ dissolution kinetics in porcine skin. Optical coherence tomography (OCT) and scanning electron microscopy (SEM) were used to study the morphology of the MNs. Poloxamer solutions with/without fluorescein sodium (FS) were prepared and investigated for its rheological properties. Selected poloxamer formulations were tested for its in situ depot formation in the micropores created by MNs, and in vitro release was evaluated using vertical Franz diffusion cells. Following in vitro release studies, confocal laser microscopy was used to study the depth of penetration of FS in the neonatal porcine skin. The barrier integrity of hairless neonatal porcine skin was assessed with transepidermal water loss (TEWL) before and after MN treatment. Fourier transform infrared spectroscopy (FTIR) was used to perform structure analysis of pure materials and synthesized MN arrays, while scanning electron microscopy (SEM) was used to study the morphology of the MNs. Figure 1 indicates the schematic illustration of poloxamer-based in situ microneedle formation, and their drug delivery and characterization.

## Materials and methods

### Materials

Gantrez® S-97 (Mw = 1500.000 Da), a copolymer of methyl vinyl ether and maleic acid polymers, was provided by Ashland (Surrey, UK). Poly (ethylene glycol) (PEG, Mw = 10,000 Da), fluorescein sodium (FS) MW 376.27 Da, polyvinylpyrrolidone (PVP K32, MW 40,000 Da; PVP K90, MW 360,000 Da), and methylene blue were all purchased from Sigma–Aldrich (Dorset, UK). Pluronic® F-127 (poloxamer 407) (MW 12500 Da), Pluronic® F-87 (poloxamer 237) (MW 7700 Da), and Pluronic® F108 (MW 14600) were purchased from BASF Chemical Company, Ludwigshafen, Germany. All other chemicals and solvents used were of pure analytical grade.

### Preparation of poloxamer solutions

Poloxamer solutions were prepared using the cold method. An appropriate amount of all poloxamers (poloxamer 407, 237, and 108) with different concentrations, i.e., 15, 20, 25, and 30% (*w*/*w*), were slowly added to cold distilled water (at 4 °C) and maintained under constant stirring to form poloxamer solutions. The poloxamer solutions were kept in the refrigerator overnight until clear solutions were formed. Combinations of these poloxamers were also prepared with the same method [39] as shown in Table 1 which indicates the feed composition ratios of poloxamer formulations and their gelation temperatures.

**Table 1:** Feed composition, gelation temperature, and viscosity of variable poloxamer formulations at 25 and 32 °C (*n* = 3)

Formulations	Sol–gel transition temperature (°C)	Viscosity (Pa s)	
		25 °C	37 °C
PF 127 *w*/*w*			
15	> 40	3.25	400.6
20	34.6	593.7	3206
25	28.5	0.07	293.5
30	25.6	485.4	1091.33
P 108 *w*/*w*			
15	> 40	0.11	0.18
20	38.3	0.09	660.3
25	33.7	0.75	9722
30	29	0.01	218.6
P 87 *w*/*w*			
15	> 40	0.02	0.08
20	> 40	0.03	2.15
25	32.8	0.04	79.24
30	28.2	0.49	935.16
P108/F87 *w*/*w*			
10/10	36.6	0.13	4.98
10/15	34.9	274.18	655.82
10/20	30.1	549.87	1003.47
10/25	24.8	0.21	26.72

### Preparation of fluorescein sodium–loaded poloxamer solutions

For the preparation of FS-loaded poloxamer solutions, FS in various concentrations was dissolved in already-prepared optimized poloxamer solutions at 4 °C maintained under constant stirring as shown in Table 2. The final FS-loaded poloxamer solutions were then kept in the refrigerator until clear solutions were formed (5–10 h).

**Table 2:** Effect of variable FS concentration on gelation temperature of optimized poloxamer formulations (*n* = 3)

		Concentration of fluorescein sodium (% *w*/*w*)				
Poloxamers (% *w*/*w*)		0	0.1	0.3	0.5	0.7
		Gelation temperature (°C)				
P108	20	38.3	38.9	39.7	40.6	41.8
P108	25	33.7	34.1	34.9	36.1	38.7
PF127	20	34.6	35	36.2	37.7	39
F 87	25	32.8	34.1	35.8	37.9	40.2
P108/87	10/10	36.6	37.4	38.5	39.3	40.5
P108/87	10/15	34.9	35.6	37.9	40.1	41.3

### Rheological analysis

The rheological properties of different poloxamer solutions were analyzed by AR-2000 rheometer (TA instrument, USA) fitted with 40-mm diameter parallel plate and circulating environment system for temperature control. The rheological characteristics of these solutions were studied using flow rheology and oscillation rheology tests in order to define viscosity at room temperature (20 °C) and at body temperature (37 ± 2 °C). In flow mode, viscosity as a function of increasing shear rate (0.1–10 1/s) was observed by conducting the continuous ramp test at 25 °C for 10 min. Temperature ramp test was performed to observe the change in viscosity along with increasing temperature (20–40 °C) at frequency of 1 rad/s and shear rate of 0.1 Pa. In oscillatory rheology, elastic and viscous moduli (G′ and G″) were evaluated with temperature ramp (20–40 °C) which in turn give sol–gel transition temperature (LCST) [41, 42].

### In vitro release study from optimized thermoresponsive poloxamer gels

The in vitro release profile of FS from in situ thermoresponsive poloxamer gels was calculated by membraneless dissolution model reported earlier [38]. From each selected FS-loaded poloxamer solution, 10 g was taken in a glass vial and equilibrated until gelation (~ 5 min) in digital shaking incubator at 37 ± 1 °C. For release study, 5 ml of phosphate-buffered saline (PBS) was poured gently on the top of the gels not to disturb the gel surface layers. The samples (2 ml) for release quantification were collected at regular time intervals until the gels remained intact for the specified time period. Each time, fresh PBS medium (2 ml) was added to the glass vials to maintain the sink conditions. The drug contents were measured using a fluorescent plate reader (BMG FLUOstar OPTIMA Microplate Reader, BMG Labtech, Ortenberg, Germany) operated at 37 °C using 493 and 520 nm as excitation and emission wavelengths, respectively.

### MN fabrication

Aqueous blends of Gantrez® S-97 and PEG10000, Gantrez® S-97 and PEG200, and PVP K32/PVP K90 were prepared and poured into laser-engineered silicone micromold to fabricate MN arrays as reported previously [14, 43, 44]. The MN arrays consisted of 361 (19 × 19) conical-shaped needles with an average height of 600 μm, with base width of 300 μm and interspacing of 50 μm perpendicular to 0.5-cm^2^ area. Briefly, 400 mg of polymer solutions was gently poured onto laser-engineered silicone micromolds and centrifuged for 20 min at 35000×*g*. MN arrays were then kept at room temperature for drying for 48 h. After complete drying, the MN arrays were removed from the molds and the sidewalls of the arrays were cut and removed with the heated scalpel blade. The MN arrays were then kept in a container and sealed with the aluminum foil until further use. Table 3 indicates the formulation composition and dimensions of MNs.

**Table 3:** MN composition, depth of penetration (DOP) in neonatal porcine skin, and moisture contents of different MNs with 19 × 19 arrays and 300-μm base widths

Sample codes	Sample composition	Weight ratio (% *w*/*w*)	Height (μm)	Average DOP (μm) *n* = 3	Moisture contents (%) *n* = 3
PG10000	Gantrez® S-97/PEG10000	10: 7.5%	600	554 ± 38.02	7.00 ± 0.03
PG200	Gantrez® S-97/PEG200	10: 7.5%	400	392 ± 7.40	4.81 ± 0.29
PVP K90:32	PVP K32/PVP K90	15: 20%	600	–	3.2 ± 0.21

## Characterization of MNs

### Determination of MN mechanical strength

The mechanical strength of fabricated dissolving MN arrays was determined by subjecting to predetermined forces for compression and skin insertion. The MN arrays were subjected to TA-XT2 Texture Analyzer (Stable Microsystems, Haselmere, UK) in compression mode by employing a range of predetermined compression forces. Briefly, MN arrays before subjecting to testing were visualized using a Leica EZ4 D digital microscope (Leica, Wetzlar, Germany). Then, MN arrays were carefully attached to the texture analyzer moveable cylindrical probe on the flat stainless-steel baseplate with the needles in downward directions. The probe of TA-XT2 analyzer was then lowered in downward direction at a speed of 0.1 mm/s until MNs touched the stainless-steel solid block and then start exerting the pre-set forces. The pre-set forces hold the MN arrays in touch with the stainless-steel solid block for 30 s and after reaching the target force the probe moved upwards at a speed of 1 mm/s. MN arrays were visualized again after force applications using the light microscope and height of the needles were determined. The change in the MN heights were determined and reported as % reduction in MN heights vs applied forces. The experiment was run in triplicates [23].

### Insertion force determination of MNs in skin tissues

TA-XT2 Texture Analyzer (Stable Microsystems, Haslemere, UK) was used to investigate the insertion force of fabricated MN formulations in neonatal porcine skin. The procedure adopted is same as previously described with minor modification for TA-XT2 Texture Analyzer. Briefly, to investigate the insertion forces, neonatal porcine skin previously obtained from stillborn piglets was immediately (less than 24 h after its birth) removed and trimmed to approximately 450–500 μm with electric dermatome (Integra Life Sciences™, Padgett Instruments, NJ, USA). The skin was then carefully shaved off to remove the remaining hairs with a disposable blade and incubated in PBS (7.4) for 1 h at 37 °C. The skin sample was then placed on a 400-μm-thick dental wax sheet (Anutex®, Kemdent Works, Swindon, UK) with its dermis upside down and mounted on wooden block for support. MN arrays were then carefully attached to the vertical probe of TA-XT2 Texture Analyzer using double-sided adhesive tape. The TA instrument was set in compression mode with the trigger force set at various predetermined required forces. The probe was then moved in a downward direction at a speed of 0.5 mm/s onto the skin. As the probe came in contact with the skin tissue, it continued to pierce the skin at the preset speed of 0.5 mm/s until the required force was reached. Upon reaching the target force, the probe then moved in an upward direction at a speed of 1 mm/s [23, 45]. Experiments were conducted in triplicates.

### Depth of MN penetration determination in skin tissues by optical coherence tomography

In order to measure and visualize the depth of penetration in neonatal porcine skin tissues, further studies were carried out using optical coherence tomography (OCT) to visualize MN insertion into neonatal porcine skin in 2D. Briefly, neonatal porcine skin, which is considered a good model for human skin in terms of hair sparseness and physical properties, was obtained from stillborn piglets and immediately (< 24 h after birth) excised and trimmed to full thickness (1000 μm). The neonatal porcine skin tissues were hydrated in PBS (pH 7.4) for 1 h incubated in a water bath at 37 °C and then dried by wiping off the solution with dry tissue paper. The hydrated skin tissues were then placed on a Styropor panel. MN arrays were then inserted in hydrated neonatal porcine skin tissues using a custom-made applicator device applying force of 20 N and immediately visualized using an VivoSight® high-resolution OCT scanner (Michelson Diagnostic Ltd., Kent, UK). The skin layers were scanned at a frame rate of up to 8B scans (2D cross-sectional scans) per second. 2D images were converted into 3D representation using an imaging software ImageJ® (National Institutes of Health, Bethesda, USA). The scale of the image files obtained was 1.0 pixels = 4 μm, thus allowing the accurate measurements of MN penetration depth [44, 46]. Experiments were run in triplicates and data was presented as (mean ± SD) 3 replicate measurements of MN penetration depth.

### In situ dissolution kinetics of MN arrays

The fate or dissolution kinetics of fabricated MN formulations were investigated in situ in dermatomed neonatal porcine skin. Briefly, neonatal skin samples were carefully shaved off by a disposable razor and then thawed in PBS (pH 7.4) for 30 min. A circular skin sample was then blotted dry with tissue paper and carefully attached to the donor compartment of the Franz diffusion cell (FDC-400 flat flange mounted on an FDCD diffusion drive console providing synchronous with 15-mm orifice diameter, and kept thermostated at 37 ± 1 °C, Crown Glass Co. Inc., Sommerville, NJ, USA) with the help of cyanoacrylate glue (Loctite Ltd., Dublin, Ireland). A piece of dental wax was then used to hold the Franz diffusion cell and to support the skin. The MN arrays were then inserted into the skin section with a custom-made applicator device (11.0-N force). The assurance of MN array insertion within the skin was made possible with the application of a circular steel weight (diameter 11 mm, 5-g mass) and held for the predetermined time intervals. At each time point, the MN arrays were removed from the neonatal skin sample, flash frozen in liquid nitrogen, and kept on storage at − 20 °C until viewing. For MN arrays, visualization Leica MZ6 dissection microscope (Leica Microsystems UK Ltd., Milton Keynes, UK) fitted with a Nikon Coolpix 950 digital camera (Nikon UK Ltd., Surrey, UK) was used. Before going for in situ dissolution study, MN heights were inspected and measured during in situ dissolution at each time point; the change in MN heights was calculated again [47]. The percent reduction in MN heights was measured and the in situ dissolution was reported as remaining MN height vs time (sec). All the measurements were made in triplicates.

### Determination of water contents of MNs

Q500 Thermogravimetric Analyzer (TA Instruments, Elstree, Herts, UK) was used for the percentage water content determination of the MN arrays. MN array samples of 5.0–10.0 mg were subjected to heating from 20 to 300 °C at a heating rate of 10 °C/min. Nitrogen flow rates of 40 ml/min (balance purge gas) and 60 ml/min (sample purge gas) were kept for all the samples. The obtained experimental data was analyzed with TA Instruments Universal Analysis 2000 software, version 44 A (TA Instruments, Elstree, Herts, UK). The experiments were run in triplicates and data reported indicates the mean ± standard deviation (SD) [23].

### Microchannels visualizations by dye-binding study

The pore formation or microchannels in neonatal porcine skin sample (3 × 3 cm) by MN arrays was evaluated using 1% *w*/*v* methylene blue dye under Leica EZ4 D digital microscope (Leica, Wetzlar, Germany). Before MN array application, the porcine skin sample was carefully shaved off using a disposable razor and then incubated in PBS (7.4) for 30 min. The skin sample surface was then blotted with the tissue paper to remove the excess of solution from the surface. The skin sample was then treated with MN arrays attached with the vertical probe of TA-XT2 analyzer. Immediately, methylene blue staining was applied involving a 5-min surface application of few drops of methylene blue solution on the treated skin followed by removal of excessive stain using sterile saline swabs and later with alcohol swabs. The treated skin sample was visualized again using Leica EZ4 D digital microscope (Leica, Wetzlar, Germany) [4]. Skin samples without the application of MN arrays were used as control and visualized using Leica EZ4 D digital microscope (Leica, Wetzlar, Germany).

### Assessment of skin integrity

The barrier integrity of neonatal porcine hairless skin was evaluated rapidly before and after poration with MN arrays by transepidermal water loss (TEWL) assessment. For TEWL determinations, porcine skin samples (3 × 3 cm) were thawed in PBS for 30 min. The barrier function of neonatal porcine hairless skin was evaluated by measuring TEWL using a VapoMeter (Delfin Technologies Ltd., Kuopio, Finland) which assess the increase of TEWL after treatment with the MN arrays. Experiments were run in triplicates and data presented indicates the means ± SD [4].

### In vitro permeation study

In vitro permeation study was conducted for FS-loaded 20% *w*/*w* PF127®-based thermoresponsive gels across the dermatomed (400 μm) neonatal porcine skin samples using vertical Franz diffusion cell setup. The drug permeation study was conducted across intact skin and MN-treated (microporated) skin samples. The Franz diffusion cells setup consisted of a receptor compartment which was filled with PBS (5 ml) at pH 7.4 with an effective permeation area of 0.64 cm^2^ stirred with magnetic bar at 600 rpm and kept thermostated to 37 ± 2 °C. First, the dermatomed non-porated intact skin samples were mounted on vertical Franz cells and kept hydrated for 1 h. The skin samples were then applied 20% *w*/*w* PF127®-based thermoresponsive gels (1000 μl) containing different FS concentrations (0.1% and 0.3%). The donor compartment and sampling arm were sealed using Parafilm®. At predetermined time intervals, samples of 500 μl were withdrawn from receptor compartment and replaced with the equal volume of PBS (pH 7.4) to maintain the sink conditions. The collected samples were sealed in aluminum foil and kept in refrigerator for drug quantification using a fluorescence plate reader [3, 23, 44]. The results were expressed as cumulative % permeation and reported as mean ± SD.

A similar procedure was adapted for evaluation of drug permeation from gels across MN-treated skin with little modifications. Briefly, the selected non-soluble hydrogel-based cross-linked MN arrays (PG10000) were inserted using the custom-made applicator at a force of application of 11 N and left in contact for 1 min using a cylindrical stainless steel of 5 g. The MN array was then removed and the microporated skin samples were then clamped between the receptor and donor compartment of the Franz cells. A 20% *w*/*w* PF127®-based thermoresponsive gels (1000 μl) containing different FS concentrations (0.1% and 0.3%) were applied as before to study permeation across microporated skin samples [3].

### Confocal laser microscopy study

The distribution of the FS was investigated in skin samples by using confocal laser microscopy. After in vitro permeation study, skin tissues were then thawed with fresh PBS solution (pH 7.4) for 15 min then blotted dry with tissue paper and kept in sample blocks containing freezing agent (OCT, Sakura Finetechnical, Tokyo, Japan) and flash frozen in liquid nitrogen. Each skin tissue sample was cryosectioned with a cryostat microtome (Richard Allan Scientific, Kalmazoo, MI, USA) into 40-μm-thick pieces and collected into respective sections onto glass slides. The skin tissue sections were then evaluated for drug distribution using confocal laser scanning microscope (Leica TCS SP2 Confocal Microscope, Leica Microsystems, UK) fitted with a Nikon Coolpix 950 digital camera (Nikon UK Ltd., Surrey, UK). A series of confocal images were extracted into 3D mode using Volocity software (PerkinElmer, UK) to observe the nature of drug distribution in skin tissues [23].

### Histology studies

The histology of neonatal porcine hairless MN-untreated and microporated (MNs treated) skin samples was observed using optical coherence tomography (OCT), i.e., VivoSight® high-resolution OCT scanner (Michelson Diagnostic Ltd., Kent, UK). The skin layers were scanned at a frame rate of up to 8B scans (2D cross-sectional scans) per second. 2D images were converted into 3D representation using an imaging software ImageJ® (National Institutes of Health, Bethesda, USA) [47].

### Structural and morphological characterization of MNs

The structural and morphological characterization of pure materials and fabricated MN arrays was performed using attenuated total reflectance (ATR) FTIR spectroscopy and scanning electron microscopy (Hitachi TM 3030 digital scanning electron microscope; JEOL Ltd., Tokyo, Japan).

### FTIR spectroscopy

The structure analysis of pure materials and synthesized MN arrays was performed using FTIR Accutrac FT/IR-4100 Series (Jasco, Essex, UK) at room temperature equipped with MIRacle™ software from 4000 to 600 cm^−1^, with a resolution of 8.0 cm^−1^. The obtained spectra indicates the result of averaging 180 scans [44, 48].

### Scanning electron microscopic analysis

SEM was performed to evaluate the morphology of the fabricated MN arrays. For SEM imaging (Hitachi TM 3030 digital scanning electron microscope; JEOL Ltd., Tokyo, Japan), the MN arrays were mounted on aluminum stubs using double-sided adhesive tape and “silver dag” and coated with gold/palladium (SC515 SEM sputter coater; Polaron, East Grinstead, UK). The morphology of fabricated MN arrays was evaluated before and after subjecting to mechanical testing by TA-XT2 Texture Analyzer (Stable Microsystems, Haslemere, UK) [48].

### Quantitative analysis

Firstly, the standard calibration curve of FS in appropriate concentration ranges (μg/ml) vs florescence absorbance was constructed. The concentration of FS in release samples was quantified using fluorescent plate reader (BMG FLUOstar OPTIMA Microplate Reader, BMG Labtech, Ortenberg, Germany) operated at 37 °C using 493 and 520 nm as excitation and emission wavelengths, respectively. For quantification of FS, the gain was set at 1000.

### Statistical analysis

The data was statistically analyzed by using Student’s *t* test and one-way ANOVA with post hoc comparisons, where appropriate. In all cases, *p* value less than 0.05 was considered significant difference. GraphPad Prism Version 4.0 (GraphPad Prism Software Inc., San Diego, CA, USA), Microsoft excel (11.0, Microsoft, 2015), and OriginPro8 were used to analyze the data.

## Results and discussions

### Sol–gel phase transition of poloxamer solutions

The sol–gel phase transition temperature of poloxamer solutions containing PF127, P108, and P87 and their combinations was confirmed by investigating their rheological properties using AR2000 rheometer. The phase transition was studied by conducting the rheological experiments in flow and oscillatory mode. In flow method, viscosity as function of increasing temperature (25–40 °C) and shear rate (0.1–10 s^−1^) were observed by conducting temperature ramp and continuous ramp test. In oscillatory rheology, elastic and viscous moduli (G′ and G″) were evaluated with temperature ramp (25–40 °C) which in turn give sol–gel transition temperature. The rheological properties of poloxamer solutions were studied to understand and confirm the thermal gelation in situ at skin temperature (i.e., 32 °C). These poloxamer solutions loaded with the drug will be deposited in the microchannels/micropores created by MN array application, which will undergo in situ sol–gel transition at skin temperature and act as drug delivery depots.

## Flow rheology

### Temperature ramp test

In temperature ramp test, viscosity over extended temperature ramp (25–40 °C) was observed to evaluate and confirm their sol–gel transition at skin temperature (32 °C). Poloxamer solutions (PF127, P108, and P87) in various concentrations were prepared in cold distilled water and stored in refrigerator until clear solutions were obtained. The poloxamer solutions were then subjected to temperature ramp test using AR-2000 rheometer (TA instrument, USA) fitted with 40-mm diameter parallel plate and circulating environment system for temperature control. Table 1 indicates the viscosity of poloxamer solutions and their combinations at room temperature and body temperature (37 °C).

Poloxamers or pluronics are triblock copolymers consisting of poly (ethylene oxide)-b-poly (propylene oxide)-b-poly (ethylene oxide) (PEO-PPO-PEO) components. In our study, we investigated a range of poloxamer grades (PF127, P108, and P87) at variable concentrations (15%, 20%, 25%, and 30% *w*/*w*) that consisted of different PEO/PPO molar ratios. Poloxamers show amphiphilic properties due to their PEO hydrophilic block and central PPO hydrophobic component. Below lower critical gelation temperature (LCGT), poloxamer aqueous solutions exist in liquid state of low viscosity due to the hydration of their hydrophilic PEO components. However, above their LCGT and at selected concentrations, their viscosity increased sharply with a slight change in temperature. The possible mechanism behind poloxamer gelation is considered to be due to the self-assembly of poloxamer micelles above their LCGT. At low temperature, the poloxamers exist as unimers in aqueous solution; however, with the increase in temperature, the unimers get assembled into micelles and packed into semisolid gelled phase. This micellization of unimers is considered to occur due to the breakage of hydrogen bonds and increased hydrophobic interaction of dehydrated PPO components with temperature increase [42].

**Fig. 2:**
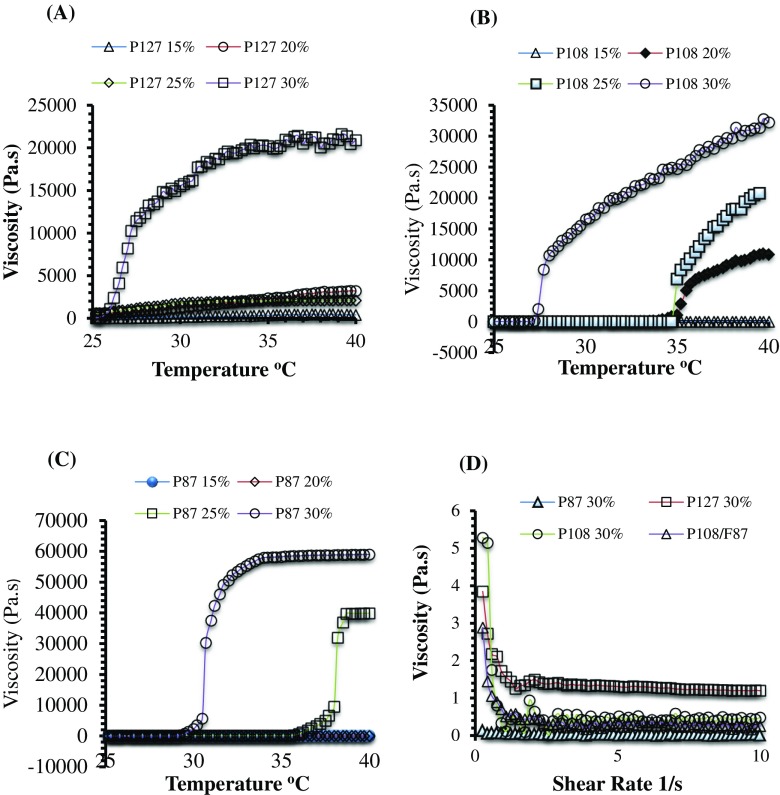
Viscosity vs temperature profile over an extended range (25–40 °C) for different poloxamers at different concentrations. **a** PF®127. **b** P108. **c** P87. **d** Viscosity as function of increasing shear rate (0.1–10 s^−1^) for 10 min at 25 °C. Each point represent the mean ± standard deviation (SD), *n* = 3

The poloxamer molecular weights (MWs) and the ratios of PPO/PEO components are the other parameters that affect the gelation properties and temperature. With increasing poloxamer concentrations, the PPO/PEO ratios increased which in turn lead to quicker micelle formation and gelation. Moreover, MW of the poloxamers is a key factor that induces the thermal gelation. As shown in Table 1, PF127 showed an increase in viscosity at a concentration of 20% *w*/*w* and temperature of 34.2 °C. However, poloxamer P108 shows a slight increase in viscosity at 20% *w*/*w* and 38.3 °C, while P87 did not show sol–gel transition at 20% *w*/*w* concentration. In our study, we have also investigated the combinations of two different poloxamers at various concentrations, MWs and different PPO/PEO ratios. As shown in Table 1, P108/87 in concentration ranges of 10/10%, 10/15%, and 10/20% induced thermal gelation in the required temperature range, i.e., 25–37 °C. Figure 2a–c indicates the change in viscosity of the poloxamers over an extended temperature range of 25–40 °C.

### Continuous ramp test

Continuous ramp test was conducted to investigate the change in viscosity with increasing shear rate (0–10 1/s) at 30 °C. All the poloxamers show a decrease in viscosity with increasing shear rate. This decrease in viscosity is suggested because of the dissociation of physical entanglements between the hydrophobic PPO components. This shows that poloxamers have thixotropic property and with increasing shear rate lead to lower cross-link density and gel disruption. Figure 2d indicates the change in viscosity of poloxamers with increasing shear rate (0.1–10 1/s) at room temperature.

### Oscillatory temperature sweep test

**Fig. 3:**
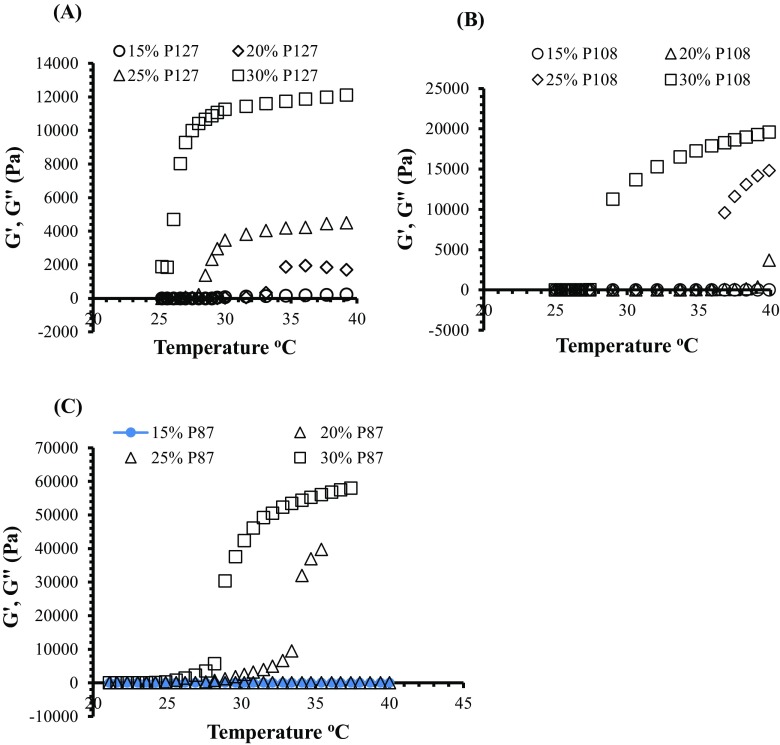
Elastic and viscous moduli (G′ and G″) evaluation with temperature over an extended range (20–40 °C) by oscillatory temperature ramp test for different poloxamers at different concentrations. **a** PF®127. **b** P108. **c** P87. Each point represents the mean ± standard deviation (SD), *n* = 3

In oscillatory temperature sweep test, elastic and viscous moduli (G′ and G″) with evaluation of temperature (25–40 °C) were investigated using AR2000 rheometer with circulating water bath. It is generally known that G′ refers to the elastic behavior of the material while G′′ indicates the viscous nature of the material. Figure 3 indicates the change in elastic and viscous moduli (G′ and G″) of poloxamer solutions with temperature change. It was observed that initially the values of viscous modulus (G″) were higher. However, with the increase in temperature, a change from sol–gel was observed with increasing elastic modulus (G′) that refers to the gel state of the poloxamer formulations. This thermal gelation or increase in elastic modulus (G′) is considered because of the dehydration of the PPO blocks with increasing temperature. Moreover, change in elastic and viscous moduli (G′ and G″) also depends upon the concentration of poloxamer solutions. As shown in Fig. 3, no significant change in the elastic modulus (G′) was observed at 15% *w*/*w* for all types of the poloxamer solutions in the required temperature range of 25–37 °C. Poloxamers (P108 and P87) did not show an increase in elastic modulus (G′) at 20% *w*/*w* as indicated in Fig. 3b, c. However, PF127 showed (Fig. 3a) an increase in elastic modulus (G′) at 20% *w*/*w* concentration. This is due to the higher hydrophobic PPO ratios in the amphiphilic structure of PF127 which quickly undergoes micellization and gel formation.

### Effect of FS loading on LCGT of poloxamer formulations

Effect of FS loading on the LCGT of optimized poloxamer formulations was investigated at various FS concentrations, mixed with pre-formulated poloxamer solutions before subjecting to rheological analysis. Table 2 indicates the effect of variable FS concentrations on CGT of the poloxamer formulations. It was observed from the results displayed in Table 2 that FS addition increased the CGT of poloxamer formulations. This is because FS is hydrophilic in nature and, with its addition, the hydrophilic interactions between the polymer–polymer and with the surrounding water molecules dominate over the hydrophobic forces, due to which relatively higher temperature is required to induce the dehydration of the PPO components and in turn thermal gelation.

### In vitro release experiments from optimized thermoresponsive poloxamer gels

**Fig. 4:**
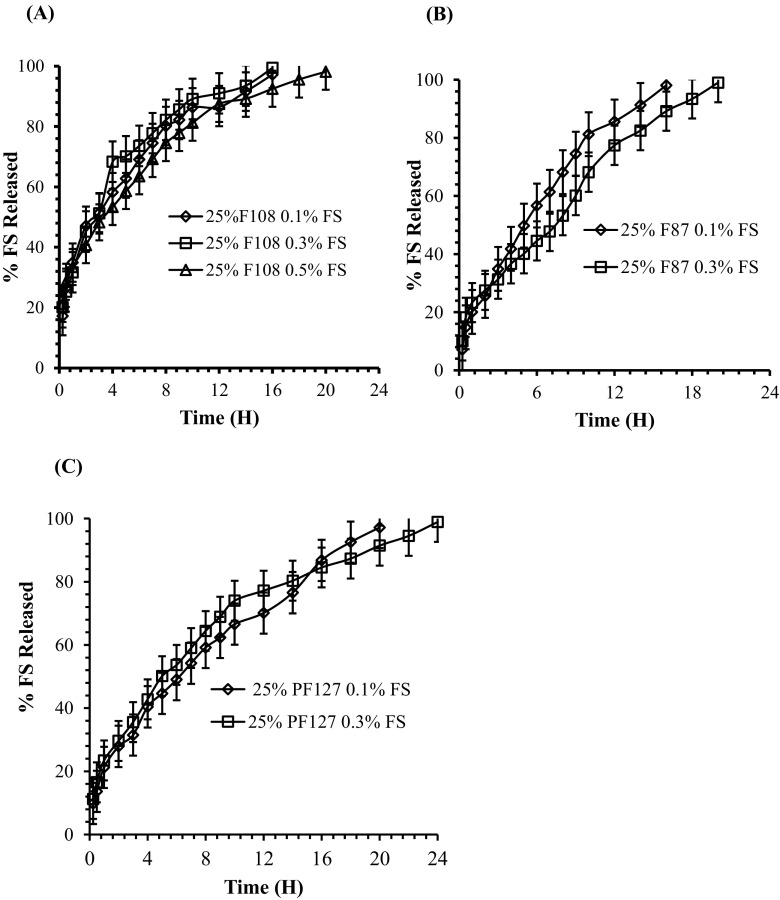
In vitro release experiments of FS from optimized poloxamer formulations at variable concentrations at 37 ± 1 °C. **a** PF108. **b** PF87. **c** PF®127. Each point represents the mean ± standard deviation (SD), *n* = 3

Release study from optimized FS-loaded poloxamer gel formulations was conducted in PBS (7.4) at 37 ± 1 °C. The FS loaded (at variable concentrations) of optimized poloxamer formulations (10 g) were transferred to a glass vial and equilibrated until gelation (~ 5 min) in digital shaking incubator at 37 ± 1 °C. In general, a biphasic release profile was observed for all poloxamer formulations containing variable FS concentrations. Initially, the poloxamer formulations showed a burst FS release within the first hour of sampling followed by sustained release. The initial burst release of FS is suggested because of the drug presence on gel surface or in the voids of the gel surface layers. Moreover, poloxamer formulations showed concentration-dependent release of FS for variable durations. Figure 4 shows the in vitro release profile of FS from all poloxamer formulations. As shown in Fig. 4a, which indicates the release profile of 25% P108 containing FS at variable concentrations (0.1%, 0.3%, and 0.5% *w*/*w*), concentration-dependent release was observed. It was found that 0.1% and 0.3% FS-loaded 25% P108 formulation provides 97 ± 0.22% and 99 ± 0.46% release during 16 h, respectively, while 0.5% FS-loaded 25% P108 formulation provides 98 ± 0.16% release for 20 h. Similarly, for poloxamer 25% P87, drug release was studied for two different concentrations (0.1% and 0.3%) and the results are displayed in Fig. 4b. Moreover, release from poloxamer PF127 was comparatively for a longer time. Figure 4c indicates the release profile of 25% PF127 containing FS at variable concentrations (0.1% and 0.3%). It was observed that 0.1% FS-loaded 25% PF127 holds the release of 97 ± 0.19% for 20 h, while 0.3% FS-loaded 25% PF127 provides 98 ± 0.92% release for 24 h. It was observed that after the maximum duration, all the poloxamer depots start dissolving due to the dominant hydrophilic interactions and the gels in the release medium did not remain intact. However, based on the in vitro release profile, it was concluded that poloxamer 25% PF127 provides the best release for comparatively longer duration and was selected to be used as the drug delivery vehicle further in in vitro permeation study.

### MN array fabrication

**Fig. 5:**
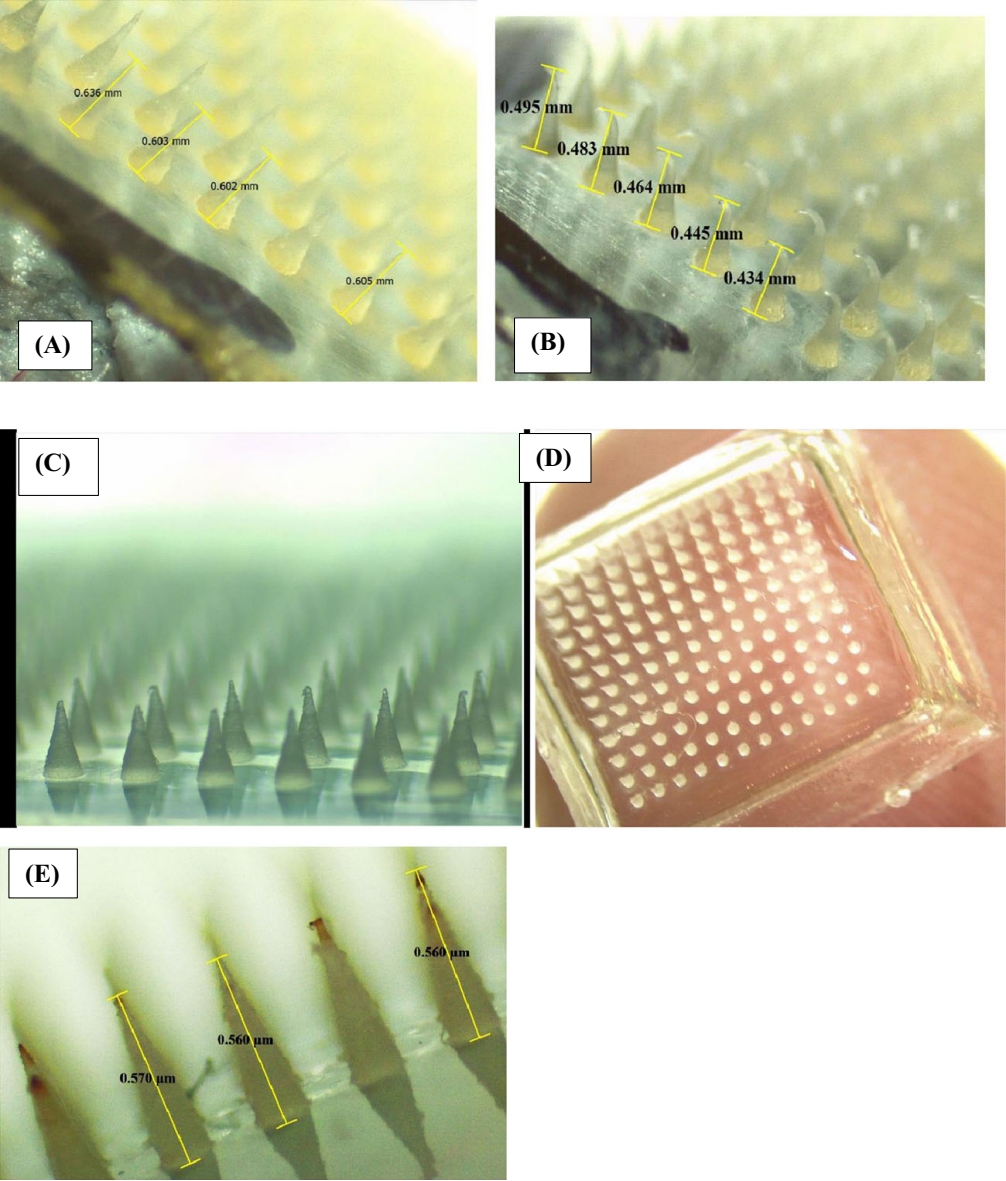
Digital microscopic images of fabricated MN arrays. **a** Gantrez® S-97 and PEG10000 (PG10000 MN arrays) before mechanical testing. **b** PG10000 MN arrays after subjecting to mechanical testing. **c** MN arrays fabricated from PVP K90 and K32 (PVP K90:32). **d** PG10000 MN arrays at lower resolution. **e** MN arrays fabricated from Gantrez® S-97 and PEG200 (PG200)

In this study, a wide range of polymers were screened in combinations and in different concentration ratios for the successful preparation of MN arrays. Table 3 summarizes the feed composition of fabricated MN array formulations. For the preparation of MN array formulations, laser-engineered silicone micromold templates with 19 × 19 needles per square grid and of two variable heights were used. Moreover, the MNs were fabricated from a variety of polymers including Gantrez® S-97, PEG10000, PEG200, PVP K32, and PVP K90 in various weight ratio combinations as shown in Table 3. However, only the MN array formulations with good mechanical strength (after mechanical testing) and higher heights (600 μm) were selected for further studies. Figure 5 indicates the digital microscopic images of fabricated MN array formulations.

## Characterization of MN arrays

### Mechanical testing of MN arrays

**Fig. 6:**
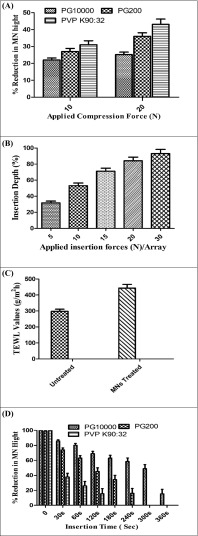
Percent reduction in MNs heights vs applied compression forces (N) representing the mechanical strength of fabricated MNs by TA texture analyzer (mean ± standard deviation (SD) of *n* = 3, *p* value = 0.246) (A). Effect of applied insertion forces (N/array) on insertion/penetration depths of fabricated MNs into neonatal porcine skin expressed as penetration depths vs applied forces (B). Untreated and MN-treated neonatal porcine skin integrity assessment by calculating transepidermal water loss (TEWL) (C). In situ dissolution kinetics of fabricated MN arrays in neonatal porcine skin expressed as % MNs height reduction vs insertion time (sec) (mean ± standard deviation (SD) of *n* = 3, *p* value = 0.545) (D)

The mechanical properties of fabricated MN array formulations (PG10000, PG200, and PVP K90:32) were tested using the Texture Analyzer. The fabricated MN arrays were subjected to two predetermined compression forces at 10 N and 20 N for 30 s. Figure 5a indicates the digital images of the PG10000 MN arrays before and after application of compression forces. It was found from the mechanical testing that the PVP-based MN arrays (PVP K90:32) showed more fragile and brittle in nature. As the compression force was applied, a progressive decrease in the MN heights was observed and was visualized using a Leica EZ4D digital microscope (Leica, Wetzlar, Germany). It was observed that MN arrays composed of Gantrez® S-97 and PEG200 (PG200) showed some good mechanical strength at all compression forces. However, the MN array formulation based on Gantrez® S-97 and PEG10000 (PG10000) showed the maximum mechanical strength against all the predetermined compression forces. Therefore, it was noted that MN arrays fabricated from high MWs PEG10000 (7.5%) and Gantrez® S-97 (10%) provide the best MN formulation with respect to their mechanical strength and creating pores in the neonatal porcine skin. Notably, no MN arrays were fractured or broken during the experiment and rather a compression of needle tips was observed. The reduction in the tips’ heights was calculated using digital microscope (Leica, Wetzlar, Germany) and the data was displayed as % reduction in MN heights vs applied compression forces as shown in Fig. 6A. Based on their good mechanical strength, PG10000 MN formulation was selected for further studies.

### Insertion force determination

The key factor in the successful applications or insertion of dissolving MN arrays to any biological tissue is their ability to withstand the applied forces. The required insertion forces for MN arrays into neonatal porcine skin were determined using texture analyzer. MN penetration into the skin tissue requires an appropriate force so that the MN arrays must have the ability to pierce the SC and create the micropores for the deposition of thermoresponsive solutions. The solutions will convert into gel at skin temperature in the microchannels and will act as drug delivery cargos. In this study, we select PG10000 MN formulation (19 × 19 square grid, 600-μm height) with total needles of 361 per 0.5-cm^2^ area owing their good mechanical strength as analyzed by mechanical testing. Figure 6B indicates the required insertion force of 5, 10, 15, 20, and 30 N per MN array vs insertion depth. It was observed from the results in Fig. 6B that with the increase in applied insertion force/array, the MN penetration or insertion efficiency also increased. The highest penetration depth was found for the higher forces, i.e., 20 N/array and 30 N/array.

### Skin integrity assessment

The barrier function of neonatal porcine skin was examined after physical treatment with MN arrays as assessed with the transepidermal water loss (TEWL). The VapoMeter (Delfin Technologies Ltd., Kuopio, Finland) was used which assesses the increase of TEWL after treatment with the devices. The VapoMeter consists of sensors which measure the percent relative humidity and convert it to a value representative of TEWL. TEWL measurement assessed the disruption degree of the skin barrier, and the base TEWL values were recorded for intact neonatal porcine skin which served as control values. The skin, being an open system, has continual water loss from its surface, but it is minimal. When the SC barrier is disrupted, the amount of water loss from skin increases. Thus, a measurable difference from the baseline is indicative of disruption of the SC and is an indirect measure of increased permeability of the skin. From the results as displayed in Fig. 6C, it was found that TEWL values increased significantly after treatment with the MN arrays which provide the evidence of disruption of SC layer and increased TEWL. Moreover, it was also observed that increased TEWL values also depend upon the larger no pores and area covered by the pores in the skin. A similar observation was made by Nguyen et al. in their study [2].

### Histology study

**Fig. 7:**
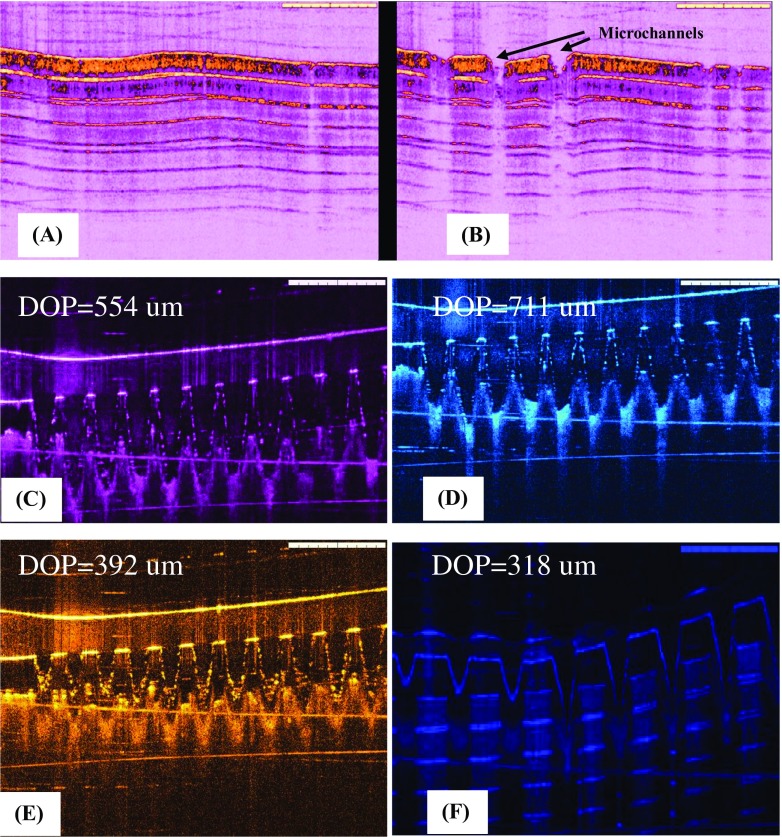
Histology of untreated porcine skin (**a**) and MN-treated neonatal porcine skin indicating the piercing of SC layer (**b**). Average depths of penetration of fabricated MN arrays into neonatal porcine skin tissues by optical coherence tomography (OCT) (**c**, **d**, **e**, and **f**). Data indicates the mean ± standard deviation (SD) of *n* = 3. DOP stands for “depths of penetration”

The histology of the neonatal porcine skin sections was evaluated using VivoSight® high-resolution OCT scanner in order to confirm the evidence of perforation with PG10000 MN arrays. Firstly, the histology of untreated skin sample having intact SC, epidermis, and dermis layers was observed with high-resolution OCT scanner. After treatment with MN arrays, the OCT images of the skin samples showed that PG10000 MN arrays effectively pierce the SC layer, penetrate the epidermis, and find their way to the dermis layer. It is evident that FS-loaded thermoresponsive poloxamer solution will flow inside these microchannels by applying on the skin, will form gel in situ at skin temperature, and will act as a delivery depot for FS. Figure 7a, b indicates the histology of the untreated and treated neonatal porcine skin samples using high-resolution OCT scanner.

### Depth of penetration by OCT imaging

The ability of MN array penetration (PG10000 and PG200) into the neonatal porcine skin tissues was evaluated using VivoSight® high-resolution OCT scanner. OCT is a non-invasive imaging technique based on light reflection, and in this study, the MN arrays were visualized in real time inserted in neonatal porcine skin. OCT imaging indicates not only the piercing of SC layer of neonatal porcine skin by MN arrays but it also shows the protruding of MN arrays into the viable epidermis and dermis layers. Figure 7c–f indicates the OCT images of in vitro insertion of two MN array formulations (PG10000 and PG200). The MN array formulations composed of Gantrez® S-97/PEG10000 and Gantrez® S-97/PEG200 owing to their good mechanical strength and non-soluble cross-linked nature showed good penetration ability in full-thickness neonatal porcine skin tissue. Table 3 indicates the original MN array heights (μm) and average depth of penetration (μm) into the neonatal porcine skin tissues. On the other hand, PVP-based MN array formulation (PVP K90:32) owing to their less mechanical properties (from mechanical testing) did not show good penetration ability. This is suggested because of the rigid and brittle nature of PVP K90:32 MN array formulation that is supposed to quickly start dissolving in skin tissues after insertion.

### In situ dissolution kinetics of MN arrays

The mechanical strength of typical MN array formulations depends upon several factors mainly polymer MW, polymer type, concentrations, moisture contents in the formulations, and preparation methods. In this study, we aim to create micropores in the skin by application of the MN arrays followed by the application of drug-loaded thermoresponsive poloxamer solution that will convert into gel and form in situ depots. To achieve this goal, the applied MN arrays should have sufficient mechanical strength to remain undissolved for a specific time period after insertion into the skin. Thus, in order to investigate the fate or in situ dissolution of fabricated MN arrays as reported in Table 3 in skin dissolution, studies were performed. Figure 6D shows the in situ dissolution kinetics of fabricated MN arrays. Our data revealed that PVP-based MN arrays (PVP K90:32) depict faster dissolution in the interstitial fluid of skin as compared to PG200 and PG10000 MN arrays. The results showed that PVP K90:32 MN arrays start dissolving in 30 s and completely dissolved in 120 s. This dissolution pattern of PVP K90:32 MN arrays confirmed the results from mechanical testing which showed the rigid and brittle nature of PVP K90:32 MN arrays with less mechanical strength. Moreover, some previous studies, in which they utilized PVP-based MNs, also report the faster dissolution in the interstitial fluid of skin [20]. Figure S1a shows the digital microscopic images of PVP K90:32 MN arrays before subjecting to in situ dissolution and during dissolution study at different time intervals. On the other hand, PG200 MNs showed some good mechanical strength as compared to PVP K90:32 MN arrays and dissolved completely in 240 s as shown in Fig. 6D. In contrast, we also found that PG10000 MN arrays start dissolution in a similar pattern in the interstitial fluid of neonatal porcine skin but showed a slower rate of dissolution of all MNs formulated. It was noted that PG10000 MN arrays started slower dissolution and remain non-soluble due to their cross-linked nature for more than 1 min. The PG10000 MNs dissolved completely in 360 s which in turn indicate their good mechanical strength due to cross-linked nature as confirmed by mechanical testing. Figure S1b shows the digital microscopic images of PG10000 MN arrays before subjecting to in situ dissolution and during dissolution study at different time intervals. From in situ dissolution study, it was concluded that due to the non-soluble cross-linked nature of PG10000 MN arrays, they can be used as tools for creating micropores during permeation study across the skin.

### Microchannels visualization

The pores or microchannels created in neonatal porcine skin followed after MN array treatment were visualized by staining the MN array–treated skin with 1% methylene blue staining. Since methylene blue, a hydrophilic dye is absorbed by the aqueous microchannels (due to the presence of interstitial fluid) created by PG10000 MNs treatment (600-μm height) with total needles of 361 per 0.5-cm^2^ area stained the channels blue. Figure S2a, b indicates the untreated porcine skin and skin sample treated with MN arrays. Methylene blue staining confirmed the poration of SC and uniformity of the pores caused by PG10000 MN arrays which shows the sharpness of the MN arrays.

### Moisture contents determination

The role of moisture contents is obvious and has high impact on the mechanical properties of MN arrays. The presence of moisture highly affects the flexibility, rigidity, and dissolution kinetics of MN arrays. The moisture contents of fabricated MN arrays were determined using thermogravimetric analysis and displayed in Table 3. It was observed that PVP-based MN arrays (PVP K90:32) showed the lowest moisture contents which reflect their rigidity and brittle nature. On the other hand, the highest moisture contents were found for PG10000 MN arrays followed by PG200 MN arrays. The presence of moisture contents also depends upon the concentration of the polymers used, their MWs, and overall environmental conditions. MN arrays were prepared in normal lab conditions; however, the moisture contents of these fabricated MN arrays can be further reduced by preparing these MN array formulations in controlled conditions (humidity and temperature) followed by drying under low-temperature environment.

### In vitro permeation study

**Fig. 8:**
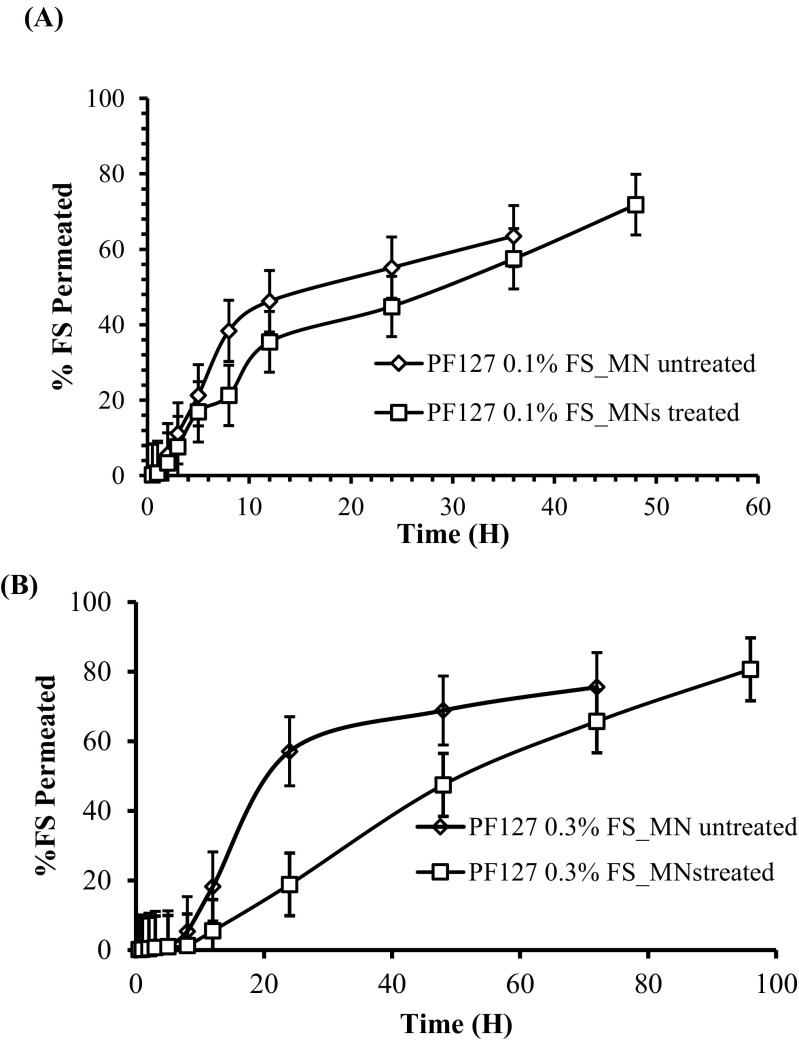
In vitro cumulative permeation profile of 0.1% FS loaded in 20% *w*/*w* PF127®-based thermoresponsive gels across MN-untreated dermatomed (400 μm) neonatal porcine skin and through microporated skin samples (**a**). Permeation profile of 0.3% FS loaded in 20% *w*/*w* PF127®-based thermoresponsive gels across MN-untreated dermatomed (400 μm) neonatal porcine skin and through microporated skin samples (**b**). Results displayed indicates the mean ± SD (*n* = 3). *p* value was < 0.01

The in vitro permeation study was conducted using vertical Franz diffusion cell to investigate the permeability of the neonatal porcine skin of FS. The permeation study was performed for 20% *w*/*w* PF127®-based thermoresponsive gels loaded with different FS concentrations (0.1% and 0.3%). The permeation was first investigated for PF127®-based thermoresponsive gels across MN-untreated porcine skin samples. Then skin samples were treated with PG10000 MN arrays owing to their non-soluble hydrogel-based cross-linked nature followed by removal of the MN arrays and application of FS-loaded PF127®-based thermoresponsive gels. The delivery of FS was investigated to confirm whether the MN array treatment followed by in situ forming depots will cause any change to delivery of FS across the skin. The accumulation of the FS in the receptor compartment of Franz diffusion cell was observed with time (Fig. 8).

The percentage cumulative permeation profile showed that a high permeation of FS and for a comparatively longer time was observed across the MN array–treated skin samples for both the concentrations used. It was observed from the release profile that 0.1% FS loaded in 20% *w*/*w* PF127®-based thermoresponsive gels provided 63 ± 0.51% permeation across MN-untreated dermatomed skin samples after 36 h, while across MN-treated skin samples, 71 ± 0.82% permeation was found until 48 h. On the other hand, it was found that 20% *w*/*w* PF127®-based thermoresponsive gels loaded with 0.3% FS showed 75 ± 0.57% permeation of FS across MN-untreated dermatomed skin samples and the delivery was saturated at 72 h. In contrast to MN-treated skin samples, percent permeation found was about 80 ± 0.69% and the permeation was held for 96 h. It is suggested that FS is hydrophilic in nature and encounter problems in diffusion across the lipophilic SC layer of the skin. However, due to its small molecular weight, FS releases from gel matrix and diffuse through the water channels present in the skin. Moreover, FS has strong binding affinity probably due to the ionic interactions and three dissociation constants with its three pKa values, i.e., 2.13, 4.44, and 6.36 respectively. PF127®-based thermoresponsive gels act as FS depot with its thermogelling property at skin temperature and provide the release of FS in sustained fashion. On the other hand, through microporated skin samples after MN array application due to their non-soluble hydrogel-based cross-linked nature, somewhat higher permeation of FS from 20% *w*/*w* PF127®-based thermoresponsive gels was found for a longer time period as compared to untreated skin samples at similar concentrations (0.3% FS). This is suggested because the SC layer of the skin is ruptured by PG10000 MN array application. The PF127®-based thermoresponsive solution loaded with FS flow inside the microchannels created by MN array application diffuse to the hydrophilic dermis layer of the skin. At skin temperature, the PF127®-based thermoresponsive solution converts into gel form and acts as reservoir for the FS in the microchannels of the skin. In this study, the permeation of FS after MN treatment was found in more controlled fashion and for a longer time duration as compared to permeation across untreated skin samples. In this study, it was confirmed for the first time that delivery of FS from in situ forming MNs in microporated sites across dermatomed porcine skin occurs in more sustained fashion. Moreover, the drug loading is not a limitation in this case as the formulation that exists is in solution form with encapsulating drug and takes MNs’ shape to form in situ depots at skin temperature as opposed to the dissolving MNs wherein drug loading can affect the microneedles’ mechanical strength.

### Distribution of FS by confocal laser microscopy

**Fig. 9:**
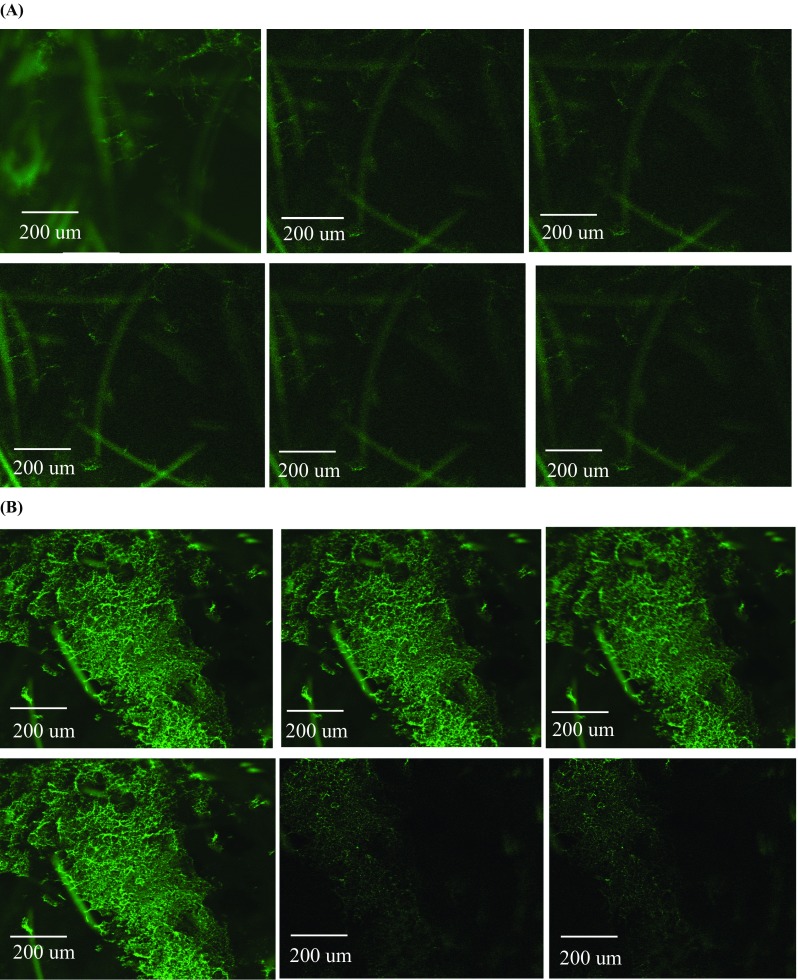
Confocal microscopic images (Z stack series) of distribution of 0.3% FS in MN-untreated neonatal porcine skin tissues after thermoresponsive poloxamers solution application during permeation study at 72 h (**a**). Distribution of 0.3% FS in MN-treated microporated neonatal porcine skin tissues after thermoresponsive poloxamers solution application during permeation study at 96 h (**b**)

In order to evaluate the nature of drug distribution, dermatomed neonatal porcine skin samples were subjected to confocal laser microscopy after termination of in vitro permeation study. The distribution of FS was tracked in both types of skin samples, i.e., MN-untreated and MN-treated samples. Confocal images of the porcine skin were observed for vertical sections and Z stack captures the images in sequence of skin sections at different depths (*z*) from the surface of the skin to determine the depth of dye permeation. It was found from confocal images that application of 20% *w*/*w* PF127®-based thermoresponsive solution containing 0.3% FS on skin surface infiltrated the skin as indicated by florescence shown in Fig. 9a. A decrease in intensity of florescence was also observed with the increase in channels’ depth. On the other hand, skin samples were microporated with PG10000 MN arrays due to their non-soluble hydrogel-based cross-linked nature followed by application of 20% *w*/*w* PF127®-based thermoresponsive solution containing 0.3% FS. In this case, the poloxamer solution diffused deep inside the channels taking the encapsulated FS. Penetration depth profile (Z stack series) shown in Fig. 9b confirmed that FS penetrated deep in the microchannels with the intense florescence. This is suggested because PG10000 MN treatment pierced the SC layer (10–15 μm thick) followed by penetration to underlying epidermis layer (50–100 μm thick) and making its way to the skin dermis. PG10000 MN treatment created pores that allowed 20% *w*/*w* PF127®-based thermoresponsive solution containing 0.3% FS to penetrate deep into the skin tissues. Confocal images confirmed the pore formation by MN treatment and permeation of FS encapsulated in 20% *w*/*w* PF127®-based thermoresponsive solution into the deeper tissues of the skin. In our study, the permeation of FS across the skin was also found by application of 20% *w*/*w* PF127®-based thermoresponsive solution containing 0.3% FS, however, with lower intensity in the deep tissues, which in turn suggest its lower permeability with MN-untreated skin.

### Chemical characterization of MNs by using FTIR spectroscopy

To evaluate the crosslinking reaction between the polymers used in the manufacturing of fabricated MN arrays, infrared spectroscopy was used. Figure S3 shows the IR spectra of the pure materials and the fabricated PG10000 MN arrays. The non-crosslinked hydrogel presents a single peak at ~ 1745 cm^−1^ that can be assigned to the acid carbonyl groups of Gantrez® S-97. In the FTIR spectra of PG10000, it shows three different carbonyl peaks. The first peak at ~ 1340 cm^−1^ can be assigned to the formation of anhydride groups between adjacent acid groups in the Gantrez® S-97 chains. The second carbonyl peak (1750 cm^−1^) indicates the ester carbonyl formed between the Gantrez® S-97 acid groups and the terminal hydroxyl groups from PEG chains. The third carbonyl peak as observed out at ~ 1490 cm^−1^ corresponds to the acid carbonyl groups. The main difference between uncross-linked Gantrez® S-97 and cross-linked Gantrez® S-97/PEG10000 is the new band that can be attributed to the new ester bonds formed between the Gantrez® S-97 acid groups and the hydroxyl groups of the PEG molecules.

### Morphological analysis by SEM

**Fig. 10:**
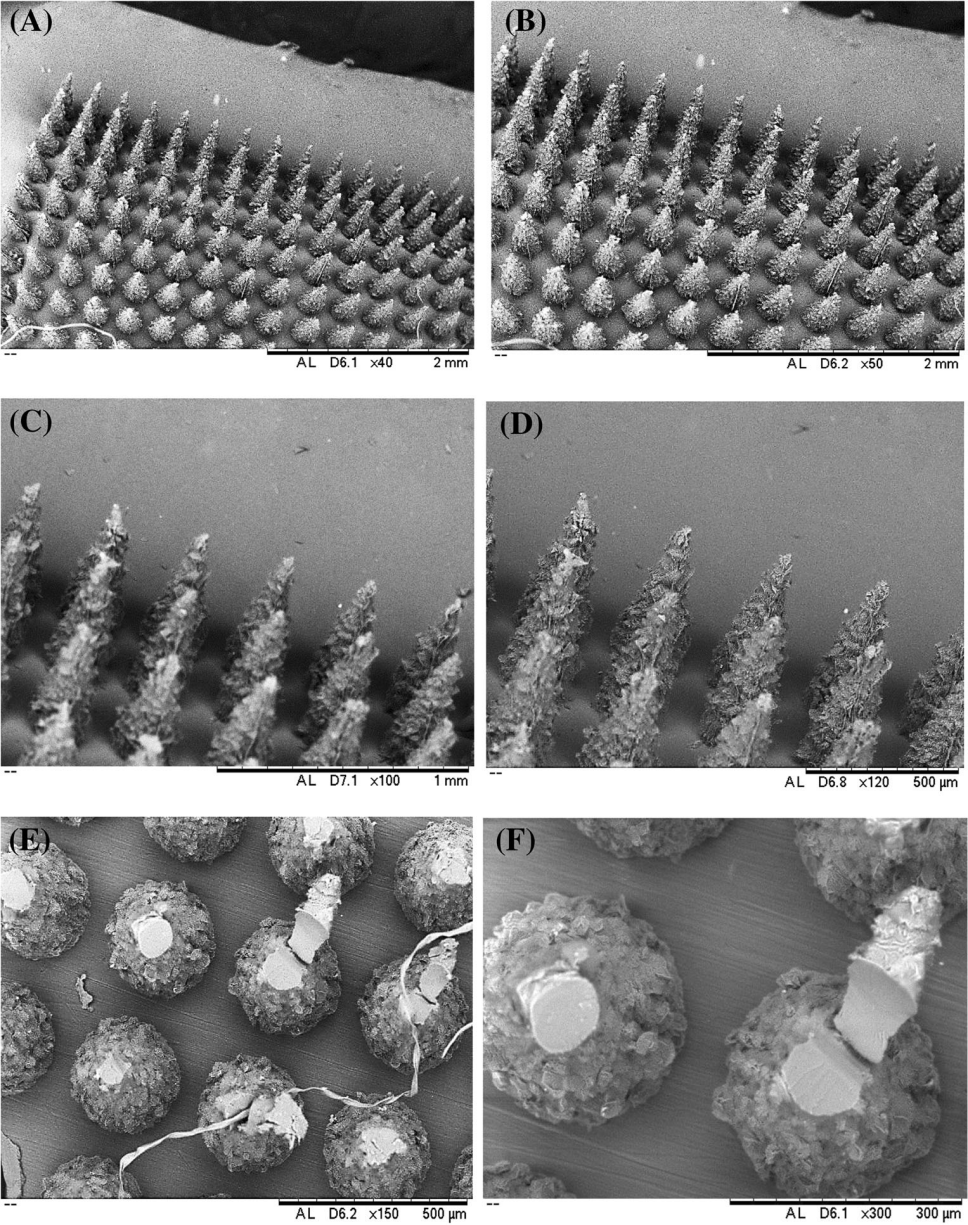
Scanning electron microscopy of PG10000 MN arrays before mechanical testing. **a** × 40 magnification. **b** × 50 magnification. **c** × 100 magnification. **d** × 120 magnification. **e** SEM analysis after mechanical testing × 150 magnification. **f** × 300 magnification

The morphology of the fabricated MNs was analyzed by SEM. The fabricated MNs were subjected to SEM analysis before and after mechanical testing of the MNs at various resolutions. From SEM analysis, it was noticeable that the needles were uniformly sharp before subjecting to the mechanical testing by TA texture analyzer and none of the needles entirely failed due to fracture or prior to mechanical testing. However, tip bending or breakage was observed for fabricated MNs after subjecting to compression forces by TA texture analyzer. Figure 10 indicates the SEM images of the fabricated MNs before and after subjecting to mechanical testing by TA texture analyzer.

## Conclusion

In this study, various poloxamer grades with different MWs and in variable concentrations were investigated for their phase transition property at skin temperature (32 °C) to form in situ depots in micropores of skin treated with MN arrays. The phase transition of poloxamers in designing MN-assisted in situ forming depots was confirmed by conducting the rheological study. These in situ forming MN-assisted depots can be loaded with drug molecules of low and high MWs and can carry the lower and higher drug concentrations across the main barrier of the skin, i.e., the *stratum corneum*. MN arrays used as a source of microporation in neonatal porcine skin were fabricated from a variety of polymer materials. The fabricated MN arrays were subjected to extensive characterization studies, i.e., mechanical strength, in situ dissolution study, insertion forces, and moisture contents, and the optimized MN array formulation was selected on their ability to withstand high compression forces required for pore creation. Dye-binding study and optical coherence tomography (OCT) showed the successful skin penetration of MNs and histology of the microporated skin. Moreover, a comparatively higher and controlled permeation of FS across MN-treated neonatal porcine skin was found as compared to untreated skin. The distribution of FS in skin tissues was successfully tracked by confocal laser microscopic analysis with higher intensity of FS in MN-treated skin tissues. Cross-linking of polymers in MN fabrication and structural confirmation was done by AT-FTIR analysis. The morphology of the fabricated MNs before and after subjecting to mechanical testing was observed by SEM analysis. Finally, it was concluded that sol–gel transition characteristic of poloxamers can be utilized to create in situ forming depots in micropores of skin treated with the MNs to provide the controlled delivery of the active pharmaceutical agent.

## Electronic supplementary material

ESM 1 (DOCX 4549 kb)

## References

[1] Donnelly RF, Singh TRR, Morrow DIJ, Woolfson AD (2012). Microneedle-mediated transdermal and intradermal drug delivery.

[2] Lee JW, Park JH, Prausnitz MR (2008). Dissolving microneedles for transdermal drug delivery. Biomaterials.

[3] Sivaraman A, Banga AK (2017). Novel in situ forming hydrogel microneedles for transdermal drug delivery. Drug Deliv Transl Res.

[4] Nguyen HX, Banga AK (2017). Fabrication, characterization and application of sugar microneedles for transdermal drug delivery. Ther Deliv.

[5] Boer M, Duchnik E, Maleszka R, Marchlewicz M (2016). Structural and biophysical characteristics of human skin in maintaining proper epidermal barrier function. Postepy Dermatol Alergol.

[6] Van Smeden J, Janssens M, Gooris GS, Bouwstra JA (2014). The important role of stratum corneum lipids for the cutaneous barrier function. Biochim Biophys Acta Mol Cell Biol Lipids.

[7] Baroni A, Buommino E, De GV, Ruocco E, Ruocco V, Wolf R (2012). Structure and function of the epidermis related to barrier properties. Clin Dermatol.

[8] Alharby YA (2016). Depot injectable atorvastatin biodegradable in situ gel : development , optimization , in vitro , and in vivo evaluation. Drug Des Devel Ther.

[9] Hong X, Wei L, Wu F, Wu Z, Chen L, Liu Z (2013). Dissolving and biodegradable microneedle technologies for transdermal sustained delivery of drug and vaccine. Drug Des Devel Ther.

[10] Peng Q, Zhang Z, Gong T, Chen G, Sun X (2012). Biomaterials a rapid-acting , long-acting insulin formulation based on a phospholipid complex loaded PHBHHx nanoparticles. Biomaterials.

[11] Banga AK (2009). Microporation applications for enhancing drug delivery. Expert Opin Drug Deliv.

[12] Banga AK (2006). New technologies to allow transdermal delivery of therapeutic proteins and small water-soluble drugs. Am J Drug Deliv.

[13] Sachdeva V, Banga AK (2011). Microneedles and their applications. Recent Pat Drug Deliv Formul.

[14] Thakur RRS, Fallows SJ, McMillan HL, Donnelly RF, Jones DS (2014). Microneedle-mediated intrascleral delivery of *in situ* forming thermoresponsive implants for sustained ocular drug delivery. J Pharm Pharmacol.

[15] Scott JA, Banga AK (2015). Cosmetic devices based on active transdermal technologies. Ther Deliv.

[16] Sullivan SP, Koutsonanos DG, del Pilar Martin M, Lee JW, Zarnitsyn V, Choi S-O, Murthy N, Compans RW, Skountzou I, Prausnitz MR (2010). Dissolving polymer microneedle patches for influenza vaccination. Nat Med.

[17] Li G, Badkar A, Nema S, Kolli CS, Banga AK (2009). In vitro transdermal delivery of therapeutic antibodies using maltose microneedles. Int J Pharm.

[18] Katikaneni S, Badkar A, Nema S, Banga AK (2009). Molecular charge mediated transport of a 13 kD protein across microporated skin. Int J Pharm.

[19] Donnelly RF, Thakur RRS, Tunney MM, Morrow DIJ, Mccarron PA (2009). Microneedle arrays allow lower microbial penetration than hypodermic needles in vitro. Pharm Res.

[20] Prausnitz MR (2004). Microneedles for transdermal drug delivery. Adv Drug Deliv Rev.

[21] Thakur RRS, Tekko IA, Al-shammari F (2016). Rapidly dissolving polymeric microneedles for minimally invasive intraocular drug delivery. Drug Deliv Transl Res.

[22] Jiang J, Gill HS, Ghate D, Mccarey BE, Patel SR, Edelhauser HF (2017). Coated microneedles for drug delivery to the eye. Cornea.

[23] Jiang J, Moore JS, Edelhauser HF, Prausnitz MR (2009). Intrascleral drug delivery to the eye using hollow microneedles. Pharm Res.

[24] Gilger BC, Abarca EM, Salmon JH, Patel S, Sciences C, Carolina N (2017). Treatment of acute posterior uveitis in a porcine model by injection of triamcinolone acetonide into the suprachoroidal space using microneedles. Physiol Pharmacol.

[25] Ita K (2015). Transdermal delivery of drugs with microneedles—potential and challenges. Pharmaceutics.

[26] Gill HS, Prausnitz MR (2007). Coated microneedles for transdermal delivery. J Control Release.

[27] Park J, Allen MG, Prausnitz MR (2005). Biodegradable polymer microneedles : fabrication , mechanics and transdermal drug delivery. J Control Release.

[28] Roxhed N, Griss P, Stemme G (2008). Membrane-sealed hollow microneedles and related administration schemes for transdermal drug delivery. Biomed Microdevices.

[29] Patel SR, Lin AS, Edelhauser HF, Prausnitz MR (2011). Suprachoroidal drug delivery to the back of the eye using hollow microneedles. Pharm Res.

[30] Bodhale DW, Nisar ÆA (2010). Structural and microfluidic analysis of hollow side-open polymeric microneedles for transdermal drug delivery applications. Microfluid Nanofluid.

[31] Berenschot JW, De Boer MJ, Yeshurun Y, Hefetz M, Illit N, MMB V (2002). Silicon micromachined hollow microneedles for transdermal liquid transfer. J Microelectromech Syst.

[32] Tuan-mahmood T, Mccrudden MTC, Torrisi BM, Mcalister E, Garland MJ, Thakur RRS (2013). European Journal of Pharmaceutical Sciences Microneedles for intradermal and transdermal drug delivery. Eur J Pharm Sci.

[33] Aoyagi S, Izumi H, Fukuda M (2008). Biodegradable polymer needle with various tip angles and consideration on insertion mechanism of mosquitos proboscis. Sensors Actuators A Phys.

[34] Donnelly RF, Singh TRR, Woolfson AD (2010). Microneedle-based drug delivery systems: microfabrication, drug delivery, and safety. Drug Deliv.

[35] Singh TRR, Tekko I, McAvoy K, McMillian, H, Jones DS, Donnelly RF. Minimally-invasive microneedles for ocular drug delivery. Expert Opin Drug Deliv. 2016;14(4):525–37. 10.1080/17425247.2016.1218460 27485251

[36] Chung HJ, Lee Y, Park TG (2008). Thermo-sensitive and biodegradable hydrogels based on stereocomplexed pluronic multi-block copolymers for controlled protein delivery. J Control Release.

[37] Moreno E, Schwartz J, Larrañeta E, Nguewa PA, Sanmartín C, Agüeros M, Irache JM, Espuelas S (2014). Thermosensitive hydrogels of poly (methyl vinyl etherco-maleic anhydride) – Pluronic® F127 copolymers for controlled protein release. Int J Pharm.

[38] Nasir F, Iqbal Z, Khan JA, Khan A, Khuda F, Ahmad L, Khan A, Khan A, Dayoo A, Roohullah (2012). Development and evaluation of diclofenac sodium thermorevesible subcutaneous drug delivery system. Int J Pharm.

[39] Derakhshandeh K, Fashi M, Seifoleslami S (2010). Thermosensitive pluronic?? Hydrogel: prolonged injectable formulation for drug abuse. Drug Des Devel Ther.

[40] Baskan T, Tuncaboylu DC, Okay O (2013). Tough interpenetrating Pluronic F127 / polyacrylic acid hydrogels. Polymer.

[41] Moreira HR, Munarin F, Gentilini R, Visai L, Granja PL, Tanzi MC, Petrini P (2014). Injectable pectin hydrogels produced by internal gelation: pH dependence of gelling and rheological properties. Carbohydr Polym.

[42] Khan S, Minhas MU, Ahmad M (2017). Self-assembled supramolecular thermoreversible β -cyclodextrin / ethylene glycol injectable hydrogels with difunctional Pluronic 127 as controlled delivery depot of curcumin . Development , characterization and in vitro evaluation. J Biomater Sci Polym Ed.

[43] Donnelly RF, Majithiya R, Raghu T, Singh R, Morrow DIJ, Garland MJ (2011). Design , optimization and characterisation of polymeric microneedle arrays prepared by a novel laser-based micromoulding technique. Pharma Res.

[44] Larrañeta E, Stewart S, Fallows SJ, Birkhäuer LL, Mccrudden MTC, Woolfson AD (2016). A facile system to evaluate in vitro drug release from dissolving microneedle arrays. Int J Pharm.

[45] Modepalli N, Shivakumar HN, Mccrudden MTC, Donnelly RF, Banga A, Murthy SN (2016). Transdermal delivery of iron using soluble microneedles : dermal kinetics and safety. J Pharm Sci.

[46] Mccrudden MTC, Zaid A, Mccrudden CM, Mcalister E, Mccarthy HO, Woolfson AD (2014). Design and physicochemical characterisation of novel dissolving polymeric microneedle arrays for transdermal delivery of high dose, low molecular weight drugs. J Control Release.

[47] Ripolin A, Quinn J, Larrañeta E, Vicente-perez EM, Barry J, Donnelly RF (2017). Successful application of large microneedle patches by human volunteers. Int J Pharm.

[48] Gomaa YA, El-khordagui LK, Garland MJ, Donnelly RF, Mcinnes F (2012). Effect of microneedle treatment on the skin permeation of a nanoencapsulated dye. J Pharm Pharmacol.

