# Supplementary material for: Evaluation of microneedles-assisted in situ depot forming poloxamer gels for sustained transdermal drug delivery
Source: Drug Deliv Transl Res. 2019 Jan 23;9(4):764–82. doi: 10.1007/s13346-019-00617-2 (PMC6606675; doi:10.1007/s13346-019-00617-2)
Supplement: Supplementary file 1 — (DOCX 4549 kb) [file 13346_2019_617_MOESM1_ESM.docx]

**Supplementary File**

**(A)**


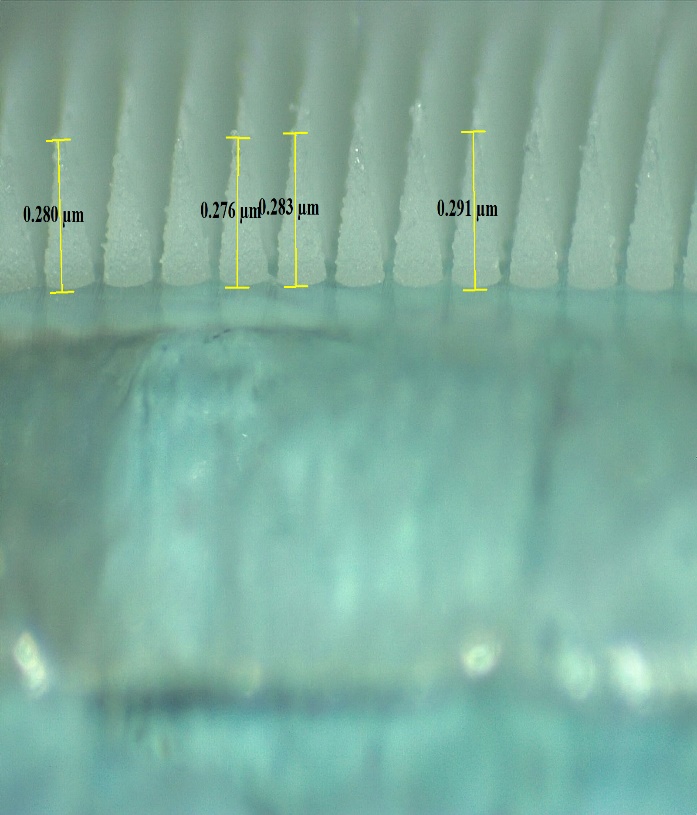

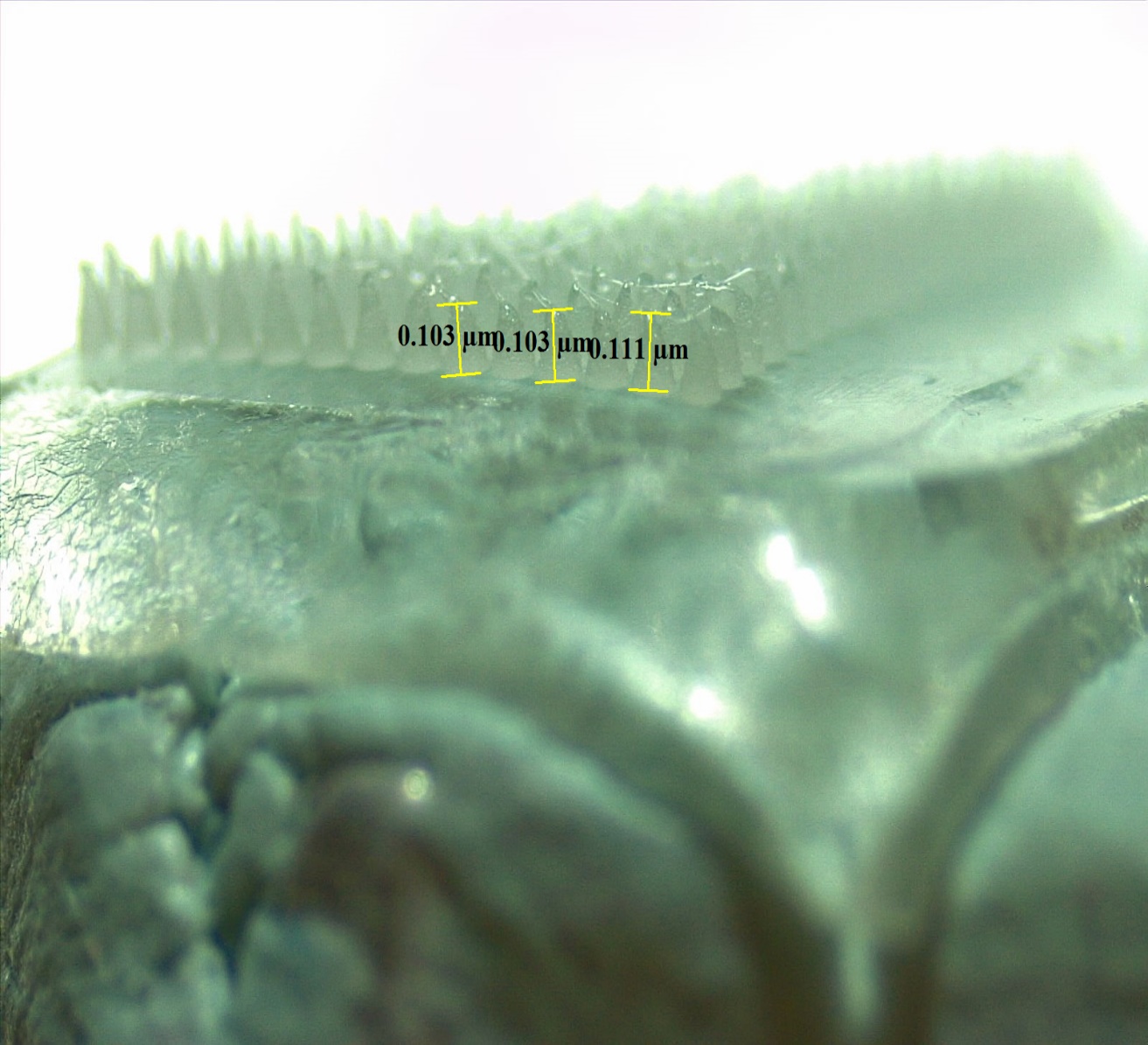


**T4= 120 sec**

**T3= 60 sec**

**T0=0 sec**

**T1= 30 sec**


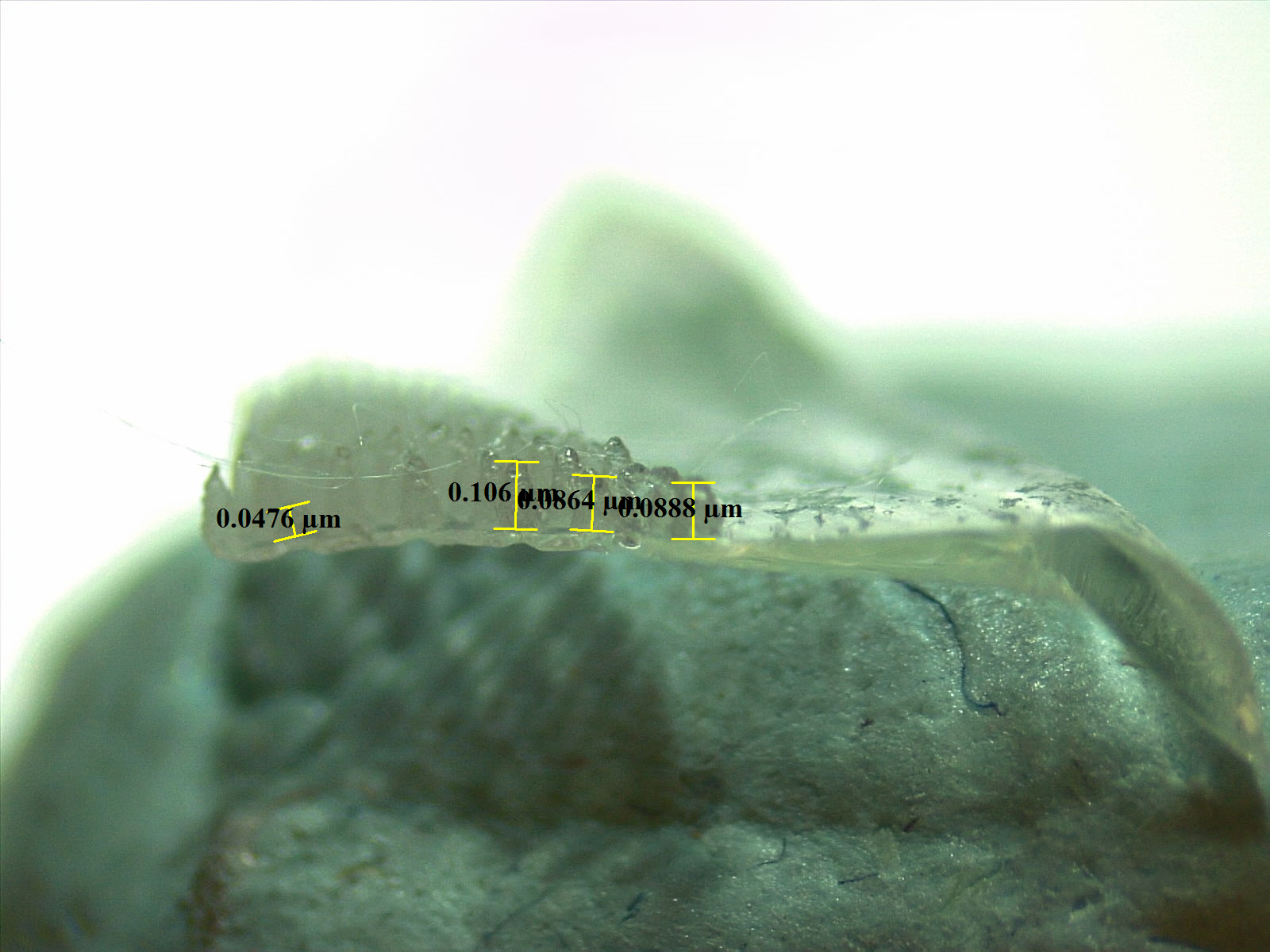

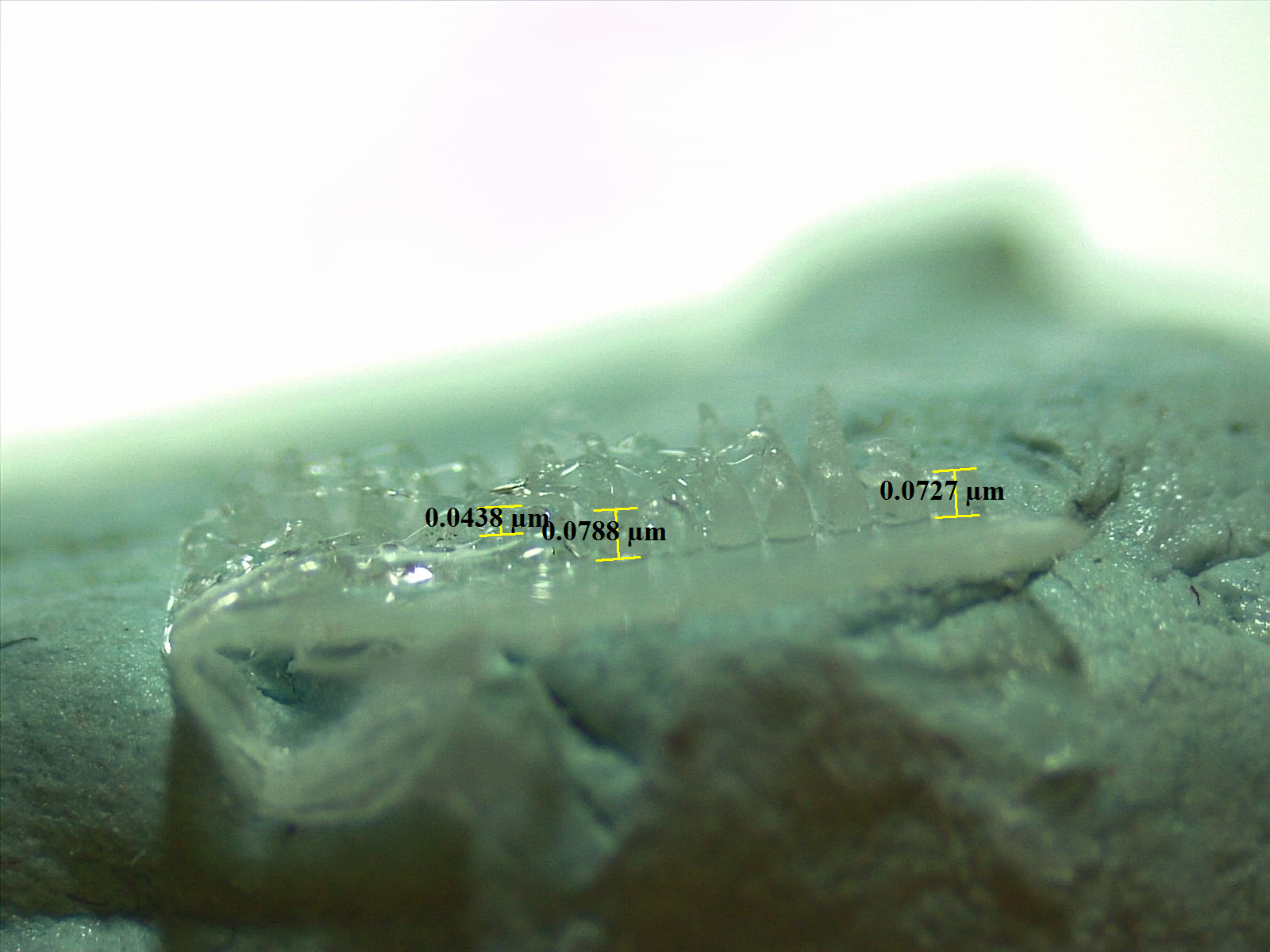


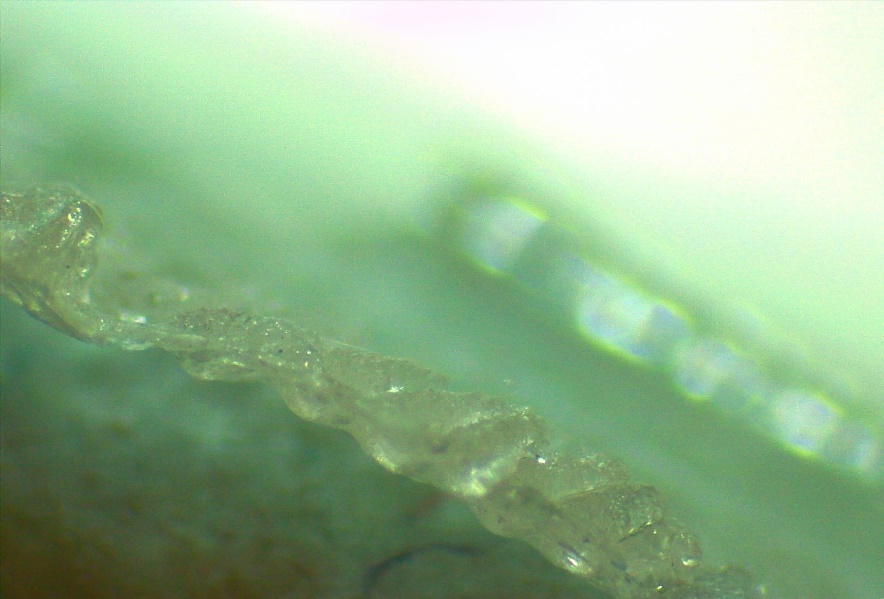


**T5= 180 sec**

**(B)**


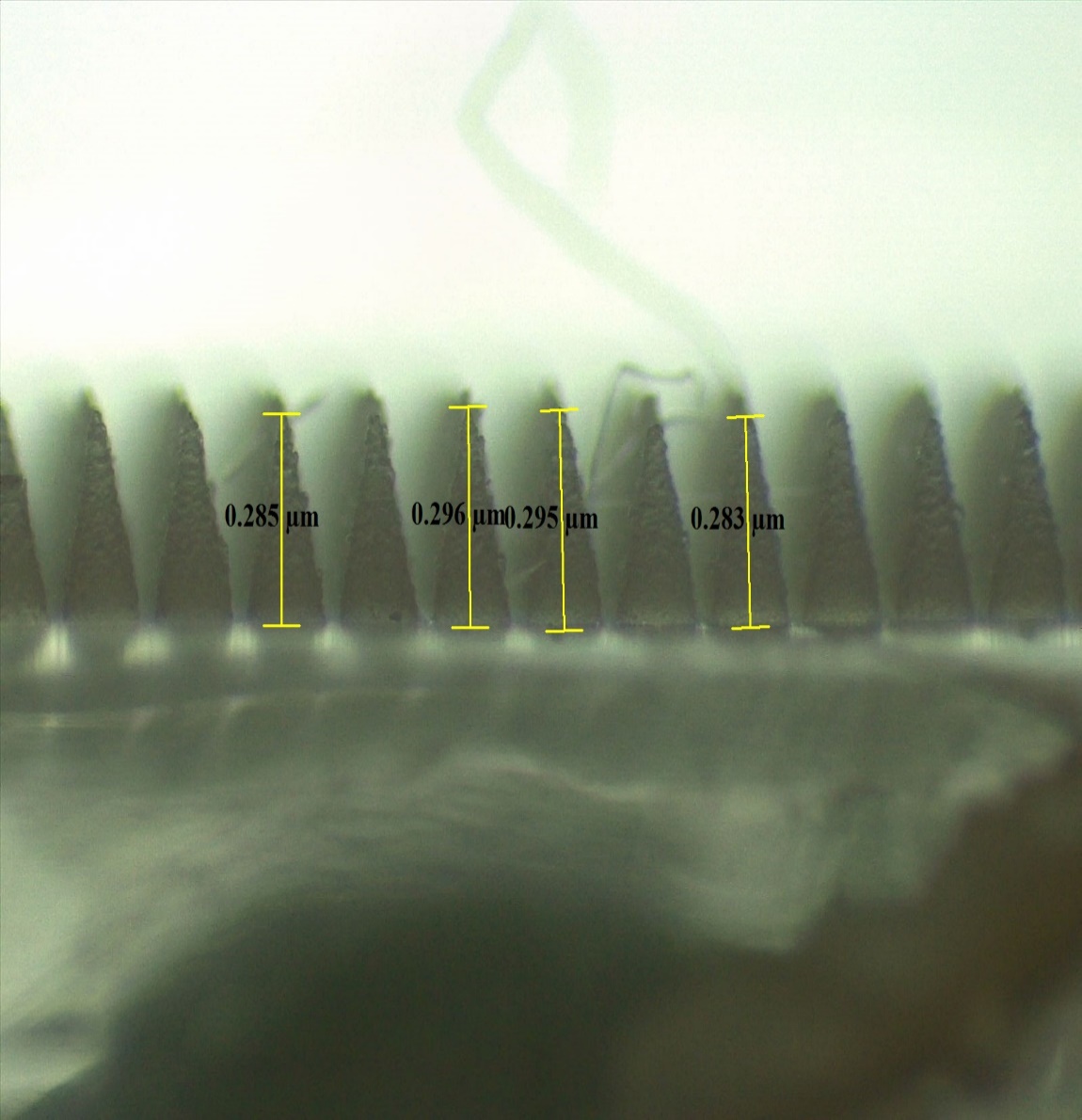

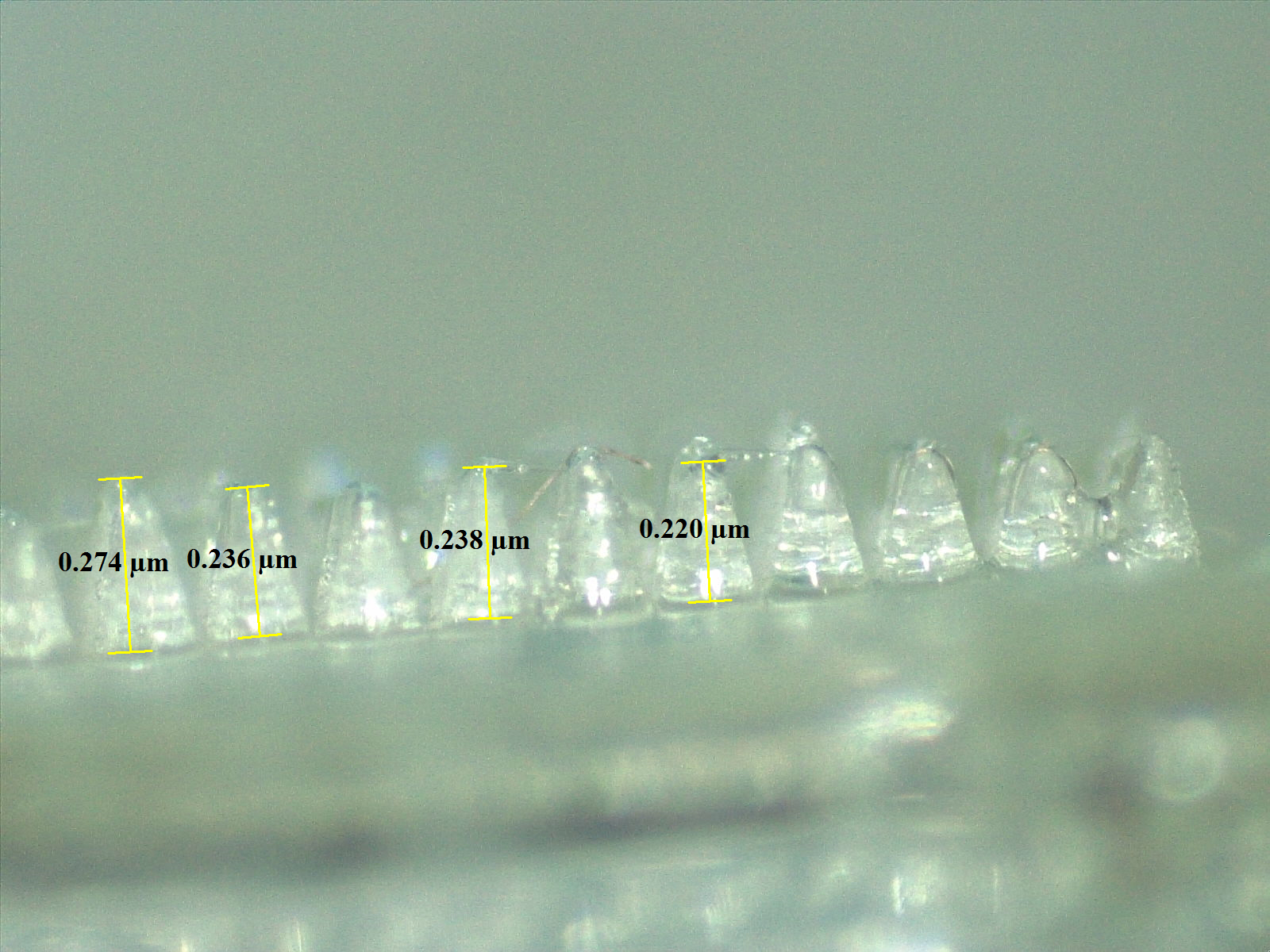


**T1= 30 sec**

**T0= 0 sec**


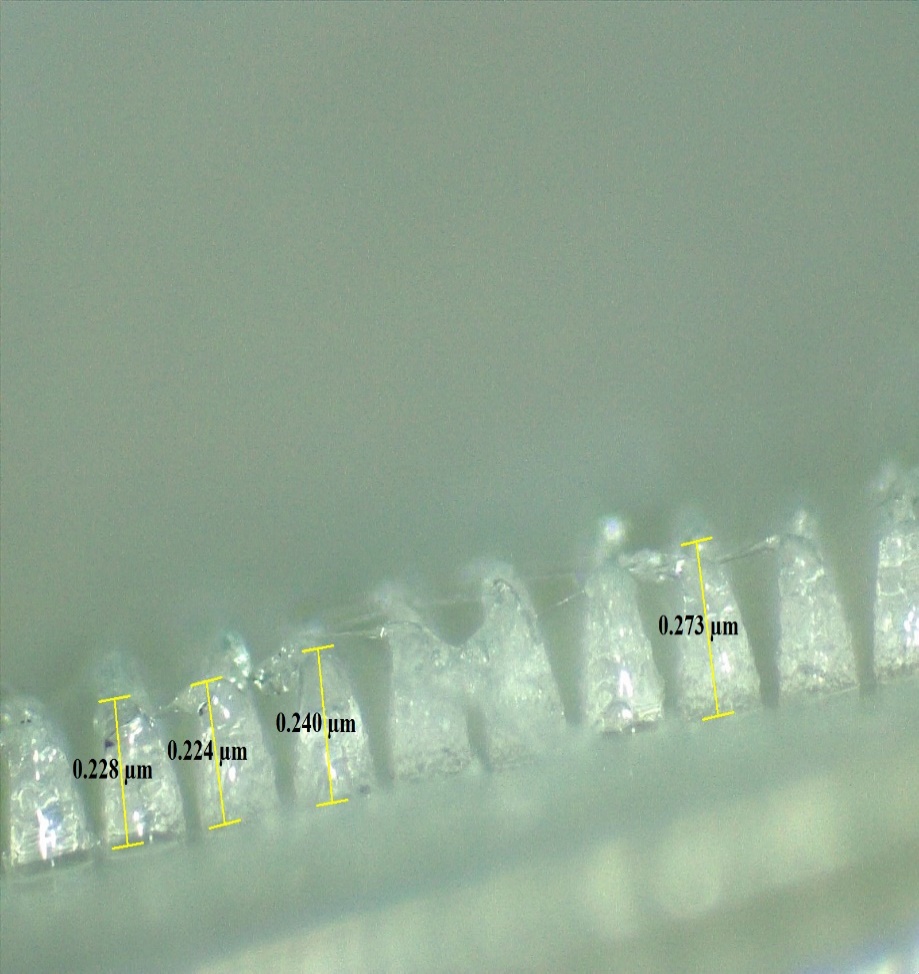

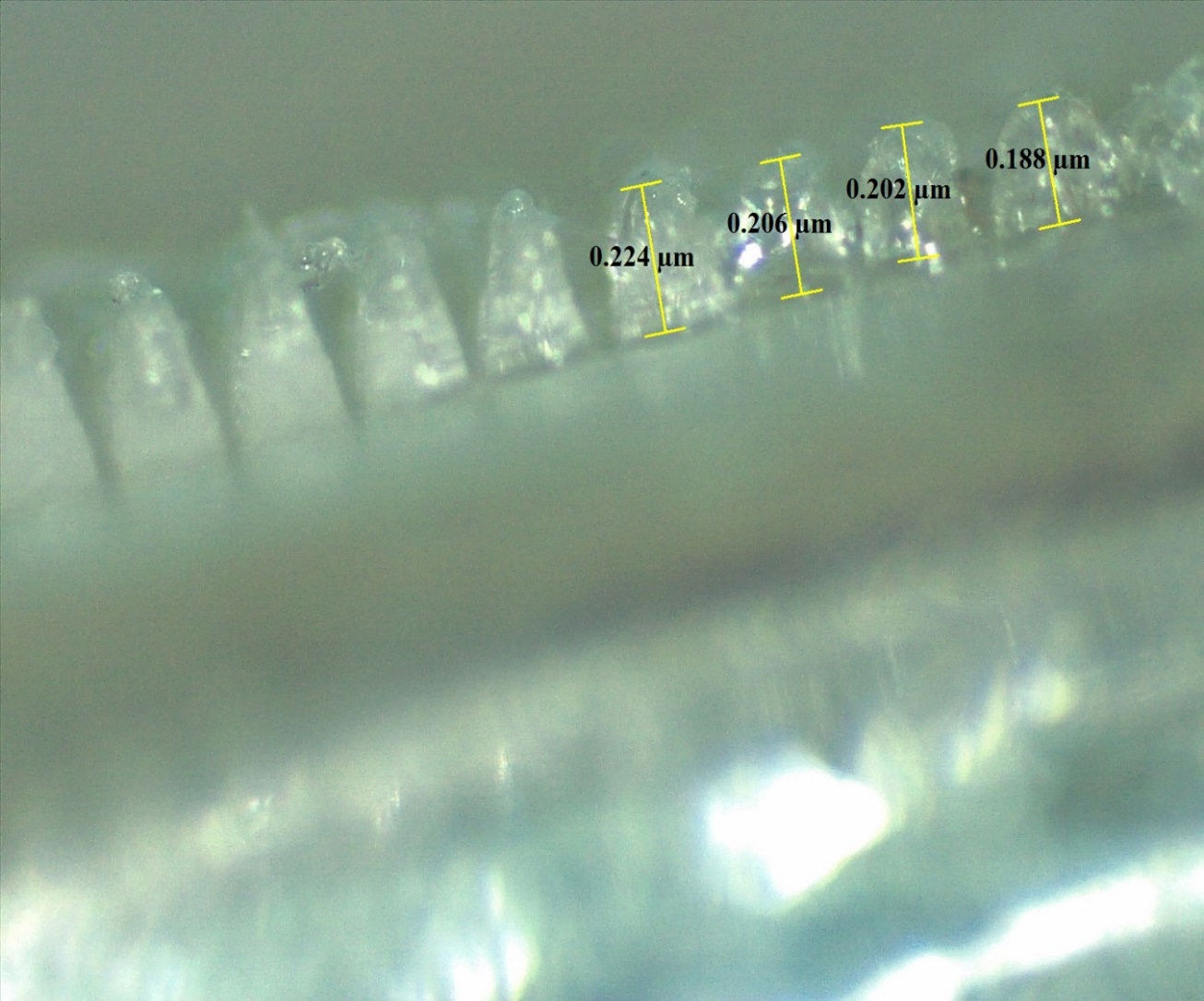


**T2= 60 sec**

**T3= 120 sec**


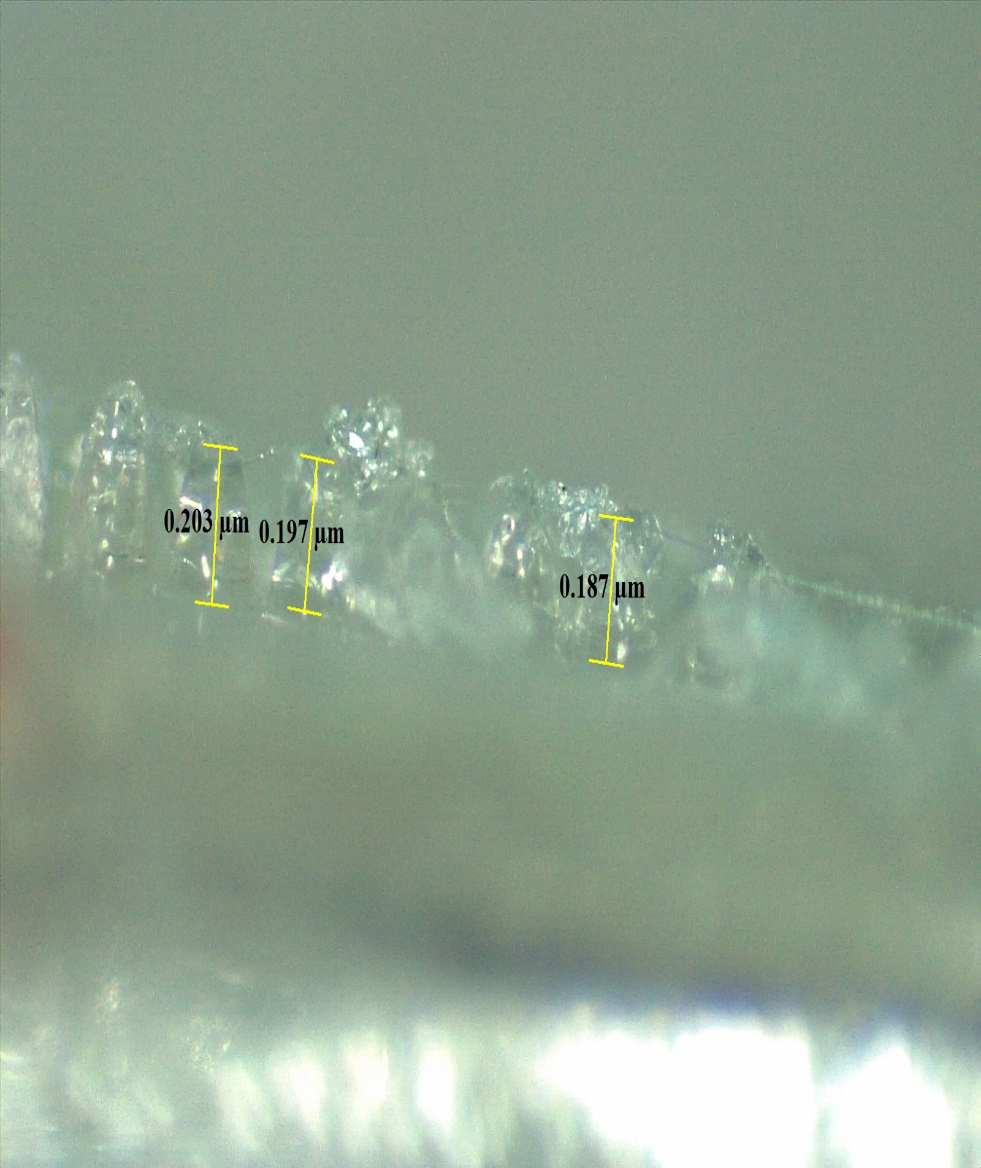

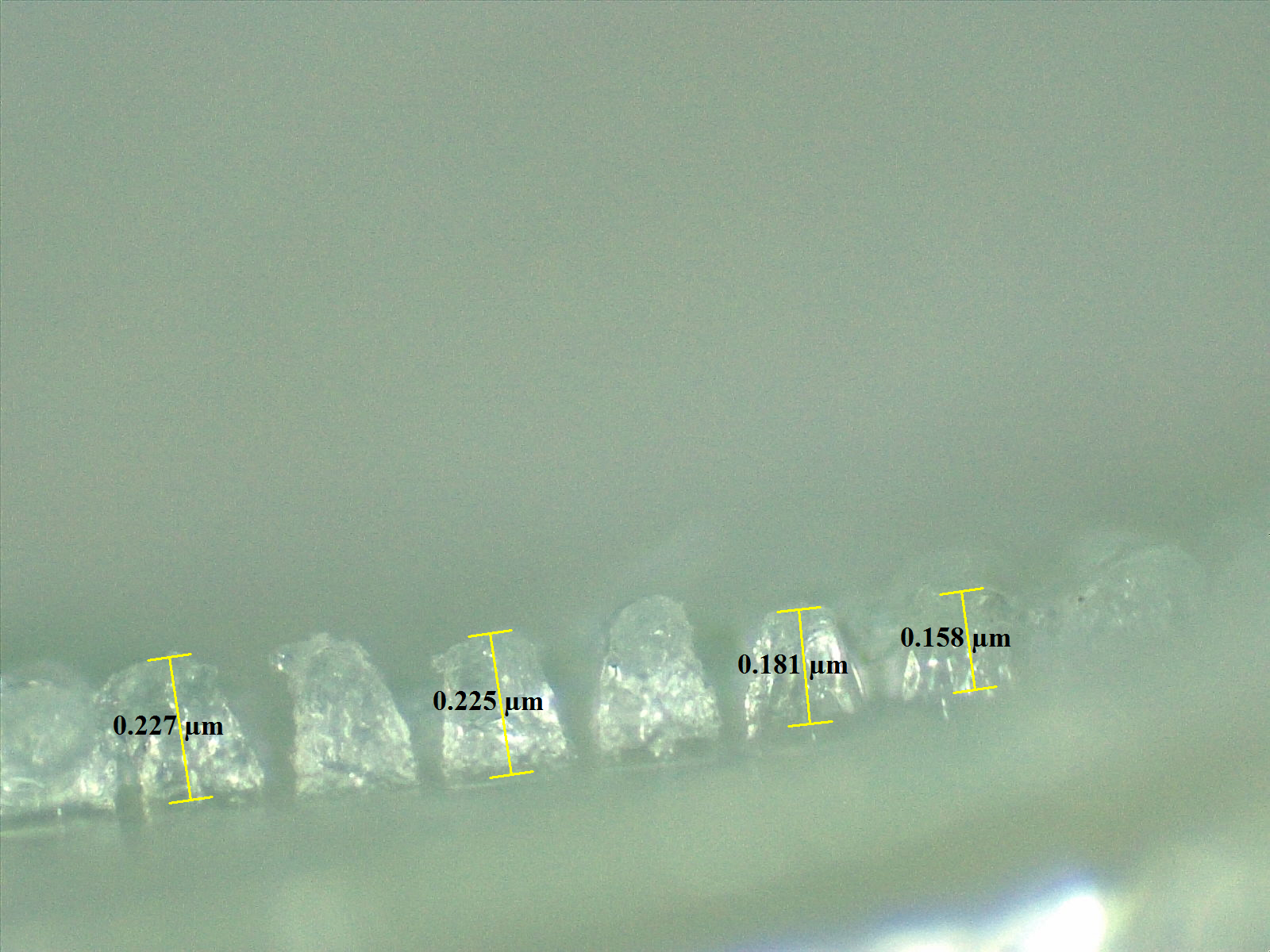


**T5= 240 sec**

**T4= 180 sec**


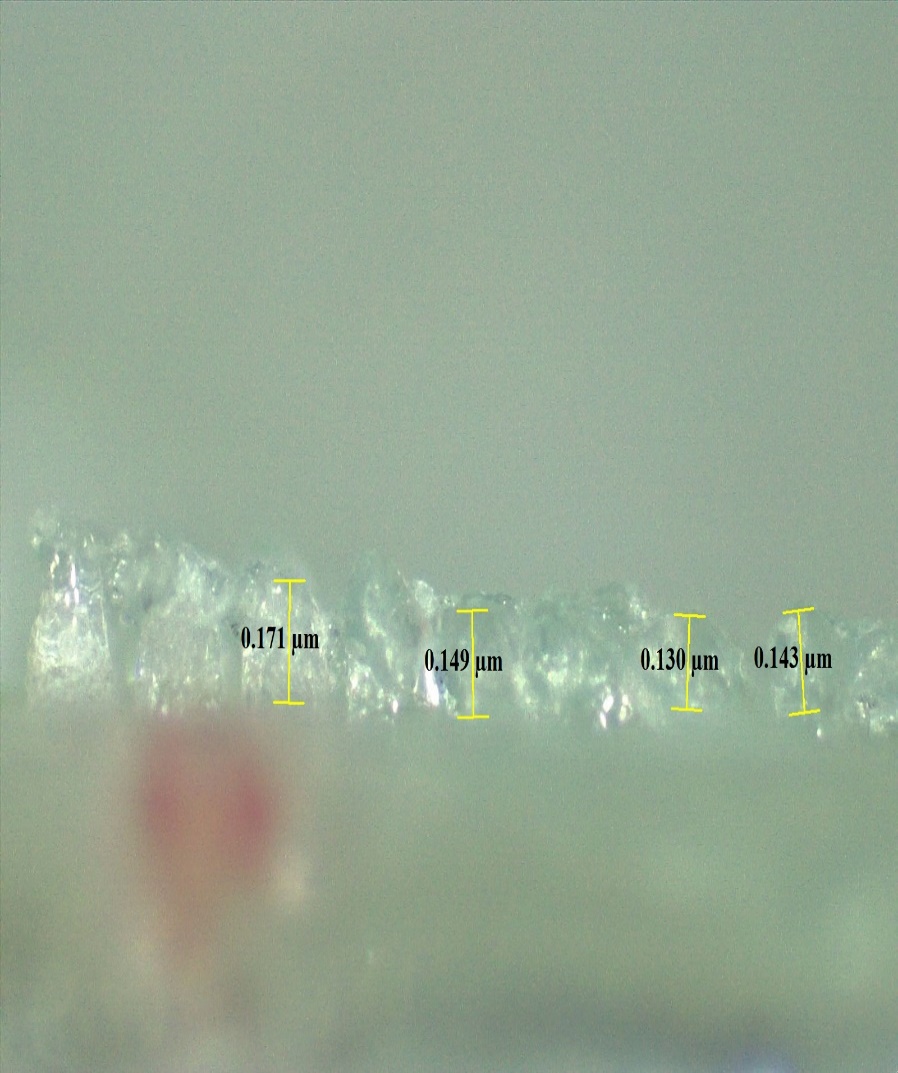

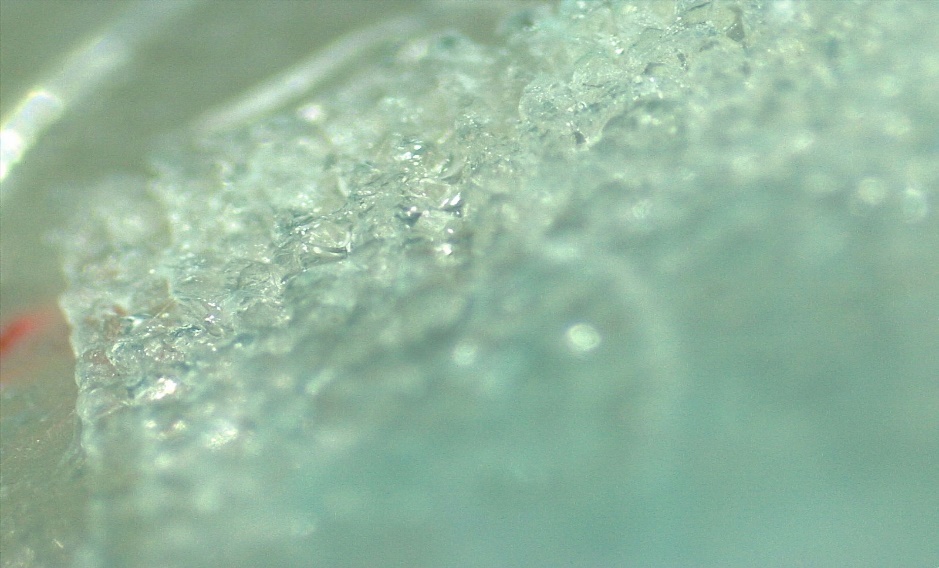


**T7= 360 sec**

**T6= 300 sec**

**Fig. S1** Digital images representing the *Insitu* dissolution kinetics of fabricated MN arrays in neonatal porcine skin at predetermined time intervals (A) PVP K90:32 MN arrays (B) PG10000 MN arrays.


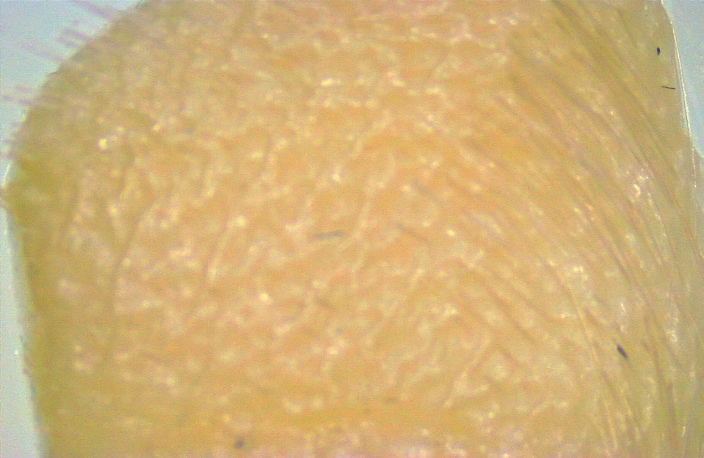

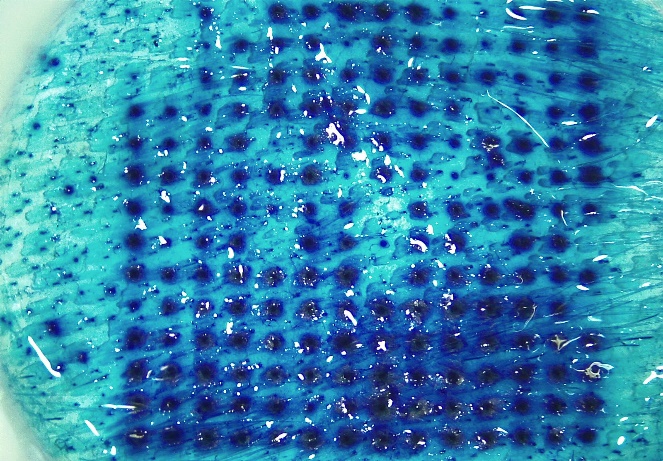


**(A)**

**(B)**

**Fig. S2** Microchannels visualization by methylene blue dye study (A) Untreated neonatal porcine skin (B) Stained neonatal porcine skin after treatment with PG10000 MN arrays (600 µm height)


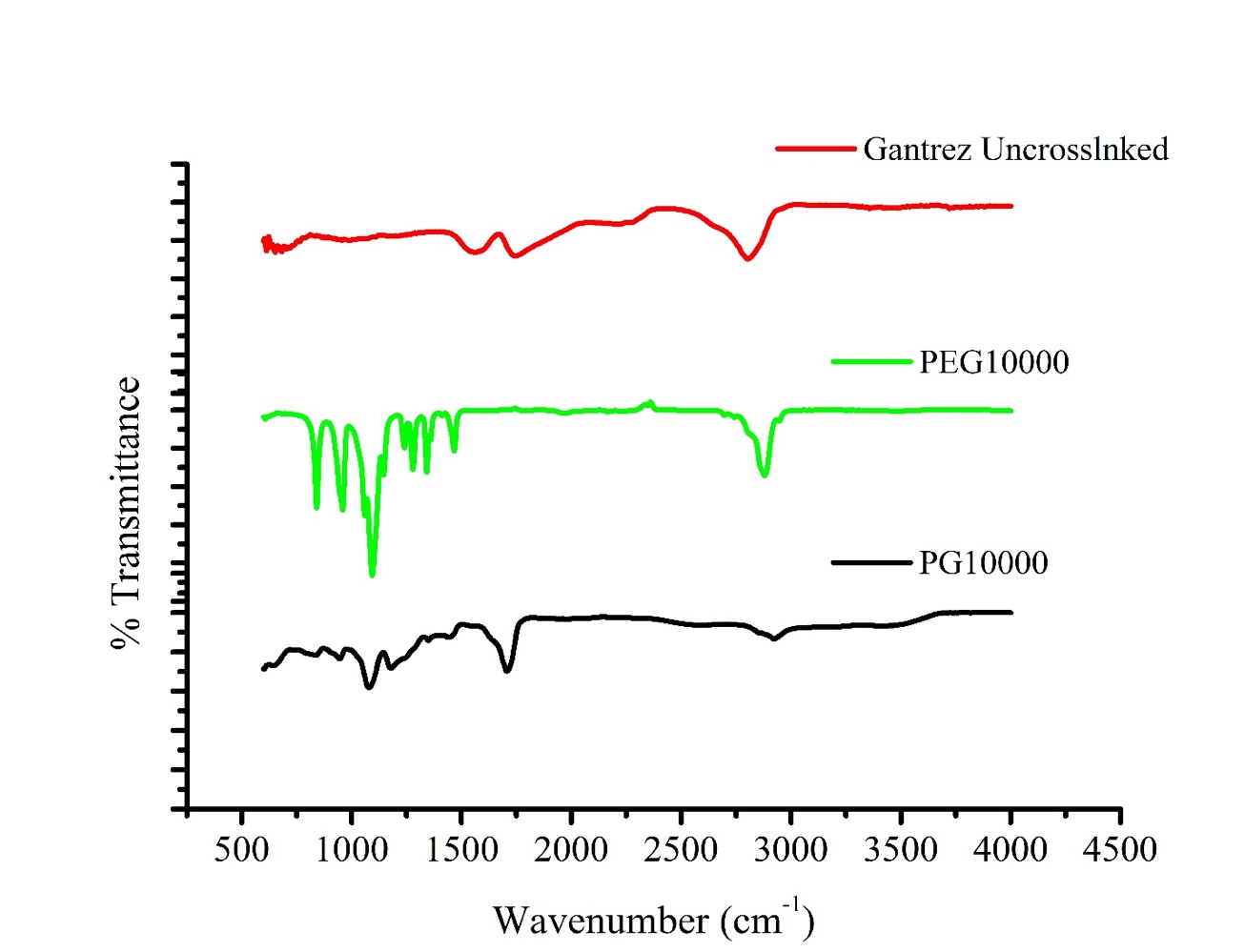


**Fig. S3** FTIR spectroscopic analysis of uncrosslinked Gantrez^®^ S-97, PEG10000 and cross-linked PG10000 MN arrays.
